# Hormone replacement therapy through the lens of epigenetic clock: estrogen signals, glycemic status, and lifespan

**DOI:** 10.1186/s13293-026-00959-w

**Published:** 2026-07-31

**Authors:** Hong Wei, Chenghua Kong, Boya Gao, Hanyu Chen, Xiaoman Dai, Yu Zhou

**Affiliations:** 1 Cadres’s Healthcare Office, Fujian Provincial Hospital, Shengli Clinical Medical College of Fujian Medical University, Fuzhou University Affiliated Provincial Hospital, Fuzhou, Fujian China; 2 Department of Clinical Pharmacy and Pharmacy Administration, School of Pharmacy, Fujian Medical University, Fuzhou, Fujian China; 3 Department of Neurology and Geriatrics, Fujian Institute of Geriatrics, Fujian Medical University Union Hospital, Fuzhou, Fujian China; 4 Fujian Key Laboratory of Molecular Neurology and Institute of Neuroscience, School of Basic Medical Sciences, Fujian Medical University, Fuzhou, Fujian China

**Keywords:** Hormone replacement therapy, Epigenetic age acceleration, Estrogen receptor, Mortality risk, Glycemic status

## Abstract

**Background:**

Epigenetic age acceleration (EAA) is a critical biomarker of aging. Hormone replacement therapy (HRT) is commonly used to alleviate age-related diseases, while glycemic status also influences the aging process. We aimed to investigate the association between estrogen and EAA, and the effects of HRT, EAA, and glycemic status on mortality.

**Methods:**

Mendelian randomization (MR) established causal relationships between genetically predicted estrogen receptor expression and EAA markers (HorvathAA, HannumAA, PhenoAA, and GrimAA). Using data from the National Health and Nutrition Examination Survey, we employed Cox proportional hazards models to analyze the association between HRT and mortality, exploring whether it was modified by EAA and glycemic status.

**Results:**

Elevated estrogen receptor α (ESR1) expression significantly decelerated PhenoAA (β -1.26; 95% CI: -2.16, -0.37; *P* = 0.005). HRT significantly correlated with a reduced risk of all-cause (HR: 0.65, 95% CI: 0.47–0.88; *P* = 0.006) and cardiovascular (HR: 0.52, 95% CI: 0.28–0.95; *P* = 0.033) mortality. Additionally, there was a significant three-way interaction among HRT, two types of EAA (HorvathAA and PhenoAA), and abnormal glucose status. Stratified analyses showed no significant interaction in the normal glucose subgroup; however, a significant antagonistic interaction emerged between HRT and both HorvathAA and PhenoAA in the abnormal glucose subgroup. Consequently, although HRT exhibited a protective main effect in patients with abnormal glucose, its survival benefits were significantly attenuated as their epigenetic aging accelerated.

**Conclusions:**

Estrogen may decelerate PhenoAA via the ESR1 pathway. In postmenopausal women, HRT is significantly associated with a reduced risk of mortality; however, this survival benefit is substantially modified by glycemic status. Future studies should evaluate whether incorporating EAA and glycemic status into HRT decision-making could optimize clinical outcomes.

**Supplementary Information:**

The online version contains supplementary material available at https://doi.org/10.1186/s13293-026-00959-w.

## Highlights

Elevated estrogen receptor α expression levels decelerate PhenoAge Acceleration.

Hormone replacement therapy (HRT) reduces all-cause and cardiovascular mortality risks.

Abnormal glucose modifies the joint effect of HRT and epigenetic aging on all-cause mortality.

HRT survival benefits in abnormal glucose diminish as epigenetic aging accelerates.

Assessing EAA and glycemic status in HRT decisions may optimize clinical outcomes.

**Supplementary Information:**

The online version contains supplementary material available at https://doi.org/10.1186/s13293-026-00959-w.

## Abstract (plain language summary)

As women get older, many experience lower estrogen levels after menopause. This can affect health in several ways, including the risk of heart disease and early death. At the same time, people do not all age at the same speed. Some may appear to be aging faster inside the body than expected for their actual age. Blood sugar problems, such as prediabetes or diabetes, may also make aging-related health risks worse. In this study, we explored whether estrogen is linked to slower biological aging and whether hormone replacement therapy (HRT) is related to a lower risk of death in postmenopausal women. We used genetic information to examine how estrogen may affect one measure of biological aging, and we also studied a large national health dataset to look at HRT use and long-term survival. Our findings suggest that estrogen may help slow one important type of biological aging through a specific estrogen-related pathway. We also found that postmenopausal women who used HRT had a lower risk of dying, including from heart-related causes. However, this benefit was not the same for everyone. In women with abnormal blood sugar levels, the helpful effect of HRT became weaker when their biological aging was more advanced. This means that blood sugar health and the speed of biological aging may both influence how well HRT works. These findings suggest that future treatment decisions about HRT may be improved by considering both blood sugar status and biological aging.

**Supplementary Information:**

The online version contains supplementary material available at https://doi.org/10.1186/s13293-026-00959-w.

## Background

Aging, as a complex and irreversible biological process, is not only a core risk factor for numerous chronic diseases but also directly leads to physiological decline and increased mortality rates [1, 2]. In the extensive research on the mechanisms of aging, epigenetic regulation, especially DNA methylation (DNAm), has been established as a crucial link connecting genotype, environment, and phenotype [3]. Based on the strong correlation between DNAm profiles and chronological age, researchers have developed a series of “epigenetic clocks”. Early first-generation clocks, including the Horvath pan-tissue clock and Hannum blood-specific clock, were primarily trained to estimate chronological age across tissues and were limited in their ability to predict health outcomes. Subsequent second-generation clocks, such as PhenoAge and GrimAge, shifted from age estimation to outcome prediction by incorporating clinical biomarkers of disease and mortality risk, thereby more directly capturing pathological aging processes [4, 5]. This, in turn, gave rise to the concept of epigenetic age acceleration (EAA), which quantifies the discrepancy between one’s epigenetic and chronological age. EAA is proven to be significantly correlated with an elevated risk of general age-related morbidity and all-cause mortality [6, 7]. Particularly in postmenopausal and older women, elevated EAA has been directly linked to specific adverse health trajectories, including compromised cardiovascular health and a significantly reduced probability of surviving to advanced age with intact mobility and cognitive function [8, 9]. These findings establish EAA as a crucial biomarker for assessing anti-aging interventions and identifying high-risk individuals.

Estrogen, particularly estradiol, is a crucial female hormone whose physiological roles extend well beyond the reproductive system to broadly impact the health of various target organs, including the skeleton and cardiovascular system [10]. However, women undergo a precipitous decline in estrogen levels during the perimenopausal and postmenopausal transitions, a hormonal shift closely linked to the onset of numerous age-related health conditions [11]. Consequently, estrogen-based hormone replacement therapy (HRT) has been widely adopted to mitigate menopausal symptoms [12, 13]. Nevertheless, its overall effect on long-term health remains a subject of debate, largely stemming from potential risks uncovered in large-scale clinical trials, such as the Women’s Health Initiative [11, 12]. Mechanistically, substantial evidence indicates that estrogen possesses anti-aging potential. Through its nuclear receptors, estrogen receptor α (ESR1) and estrogen receptor β (ESR2), it modulates gene transcription to suppress oxidative stress, enhance mitochondrial function, and regulate inflammatory responses [10, 14]. Although these mechanisms are implicated in aging, prior epigenetic research has predominantly focused on endogenous estrogen, and evidence concerning exogenous HRT remains limited. For instance, Levine et al. observed a decelerated epigenetic aging associated with HRT use exclusively in buccal epithelium [15]. To address these limitations, our study provides a comprehensive analysis of the impact of HRT on epigenetic aging.

Further research has emphasized the profound interplay between metabolic health and epigenetic aging. For instance, research by Miao et al. found that metabolic disorders, such as glycemic abnormalities, are one of the key upstream factors driving the acceleration of epigenetic clocks (like GrimAge and DunedinPACE) [16]. Building upon this foundation, work by Wu et al. validated the clinical predictive utility of these clocks, demonstrating that epigenetic aging metrics serve as robust biomarkers for all-cause and cardiovascular mortality in individuals with diabetes and pre-diabetes [17]. Therefore, accounting for metabolic status, such as blood glucose level, is essential for accurately elucidating the independent effects of estrogen on biological aging.

This study employs a multi-level analytical approach to systematically elucidate the complex interrelationships among estrogen signaling, epigenetic aging, glycemic status, and mortality risk. We propose two primary hypotheses: (1) genetically predicted higher expression of estrogen receptors (ESR1 and ESR2) causally attenuates EAA; and (2) glycemic status acts as a critical moderator in the association pathway from HRT to EAA, and ultimately to mortality. To test these hypotheses, we first implemented a Mendelian randomization (MR) approach to investigate the potential causal impact of estrogen receptor expression on various epigenetic clocks. Subsequently, to bridge our genetic findings with clinical relevance, we assessed the associations of HRT with epigenetic aging, all-cause and cardiovascular mortality using data from the National Health and Nutrition Examination Survey (NHANES). Finally, we conducted a three-way interaction analysis and a stratified analysis to clearly examine whether glycemic status would affect the combined effect of HRT and EAA on the risk of death. Importantly, while MR enables causal inference at the genetic level, the NHANES analyses are observational and intended to examine associations rather than causal effects of HRT.

## Methods

### MR study design and data sources

We implemented the drug-target MR approach to assess causality [18]. Causal inference is valid provided three core assumptions are met: (i) the instrumental variables (IVs) are robustly associated with the estrogen receptors; (ii) the IVs are not associated with any confounders; and (iii) the IVs affect the EAA exclusively through the estrogen receptors. We followed the Strengthening the Reporting of Observational Studies in Epidemiology MR reporting guidelines [19].

Summary statistics for expression quantitative trait loci (eQTL) associated with ESR1 and ESR2 expression in blood were obtained from the eQTLGen consortium [20]. This study comprised 31,684 individuals of European ancestry across 37 cohorts. Data for EAA was sourced from a genome-wide association study (GWAS) meta-analysis conducted by McCartney et al. [21], which included 6,195 individuals of African American descent. Four EAA biomarkers (HorvathAA, HannumAA, PhenoAA, and GrimAA) were quantified as the residuals from the regression of DNAm age onto chronological age. Data sources are listed in Table S1.

### IVs selection and MR analysis

A rigorous quality control protocol was applied to select valid IVs. First, cis-eQTLs associated with blood ESR1 or ESR2 expression at a genome-wide significance level (*P* < 5 × 10⁻⁸) were selected as potential instruments. To ensure independence, these single nucleotide polymorphisms (SNPs) were subsequently pruned for linkage disequilibrium using a threshold of r² < 0.3 within a 500 kb window. Instrument strength was assessed using the F-statistic, and only SNPs with F > 10 were retained to ensure sufficient instrument validity [22]. The primary causal estimates were derived using the multiplicative-random-effects inverse variance weighted (IVW-mre) method. The IVW-mre method yields the most precise estimate, assuming all genetic variants are valid instruments [22]. The weighted median method [23] and MR-Egger regression method [24] were used as complementary analyses. The effect estimate (β) represents the standard deviation (SD) change in EAA per 1-SD increase in genetically predicted estrogen receptor expression.

### Sensitivity analysis

**Fig. 1:**
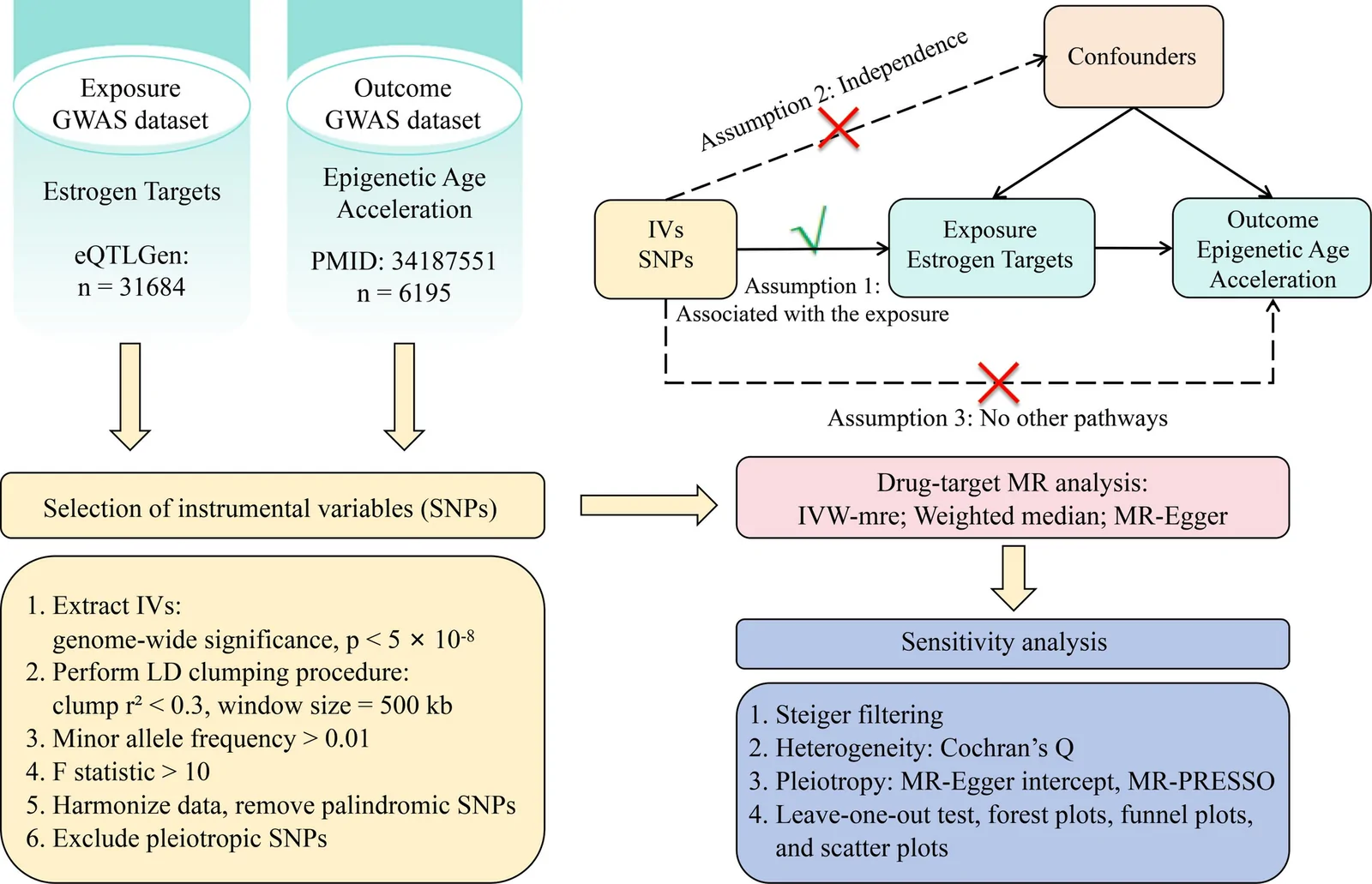
Flowchart of Mendelian randomization analysis

To minimize potential reverse causality, we applied Steiger filtering [25]. Heterogeneity was evaluated using Cochran’s Q test [26], while directional horizontal pleiotropy was assessed via the MR-Egger regression intercept [24]. We further utilized the MR-Pleiotropy RESidual Sum and Outlier (MR-PRESSO) test to identify and correct for potential outliers [27]. A leave-one-out analysis was performed to examine whether any single SNP excessively influenced the overall causal estimates. Results were visualized using forest plots, funnel plots, and scatter plots. To account for multiple testing, we applied a Bonferroni-corrected significance threshold of *P* < 0.006 (0.05/8) [28]. All analyses were conducted using the “TwoSampleMR” package (version 0.6.8) in R software (version 4.4.1). The MR workflow is depicted in Fig. 1.

### NHANES study design and population

**Fig. 2:**
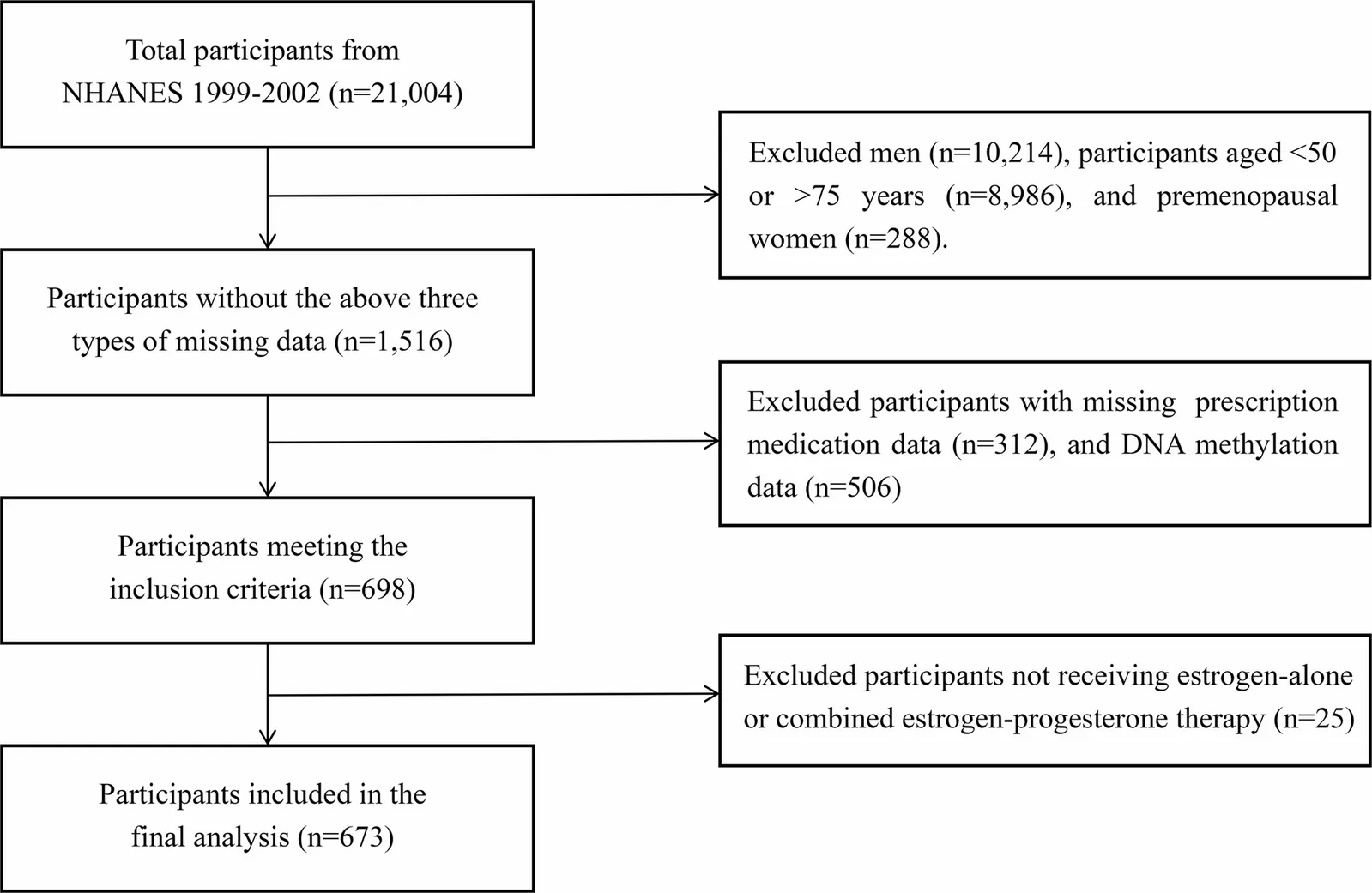
Participant selection diagram in NHANES 1999–2002

NHANES is a national cross-sectional survey designed to obtain a representative sample of the non-institutionalized U.S. population; however, because our analyses did not incorporate its complex sampling design, our findings only represent exploratory associations within the current sample rather than national estimates. This study utilized data from the NHANES 1999–2002 cycles. In this period, NHANES researchers randomly selected a portion of participants aged 50 or older to undergo DNAm testing. Based on this population, we applied prespecified screening criteria and excluded men, individuals older than 75 years, premenopausal women, and participants with missing information on prescription drug use. Menopause was defined by the presence of a follicle-stimulating hormone level ≥ 40 IU/L, a natural cessation of menstruation for at least 12 months, or a history of hysterectomy or bilateral oophorectomy. Women over 75 were excluded for two main reasons. First, NHANES age top-coding prevents the accurate calculation of EAA. Second, this exclusion minimizes confounding from age-related frailty and high mortality risks, which could obscure the studied associations. The screening process is detailed in Fig. 2.

### Variable measurement and processing

Estrogen use status was ascertained from prescription drug data collected during NHANES household interviews. Researchers recorded all prescription medications used by participants in the preceding month and classified them using National Drug Codes, with code 1034 representing estrogen/progestin therapies. Participants were subsequently categorized into two groups: the “HRT group” (those using estrogen-only or combined estrogen-progestin therapy) and the “non-HRT group”. DNA was extracted from whole blood samples collected from participants. Five epigenetic age biomarkers were calculated using processed and standardized DNAm data. EAA was derived as the residuals from linear regression of each epigenetic age on chronological age [29], generating HorvathAA, HannumAA, PhenoAA, GrimAA, and Grim2AA (an enhanced version of GrimAA [30]). For subsequent analyses, these EAA variables were standardized to evaluate the effects per 1-SD change. The primary outcomes were all-cause mortality and cardiovascular mortality, obtained through linkage with the National Death Index public records, with follow-up data up to December 31, 2019, and a median follow-up time of over 17 years. Covariates included age, race, education level, poverty income ratio, body mass index, smoking status, alcohol consumption, glycemic status, hypertension, hyperlipidemia, cardiovascular disease, chronic kidney disease, and cancer. Among these, glycemic status was categorized into normal glucose and abnormal glucose, with abnormal glucose further comprising prediabetes and diabetes. Prediabetes was defined by meeting any of the following criteria: a self-reported diagnosis of prediabetes in the questionnaire, a fasting plasma glucose (FPG) level of 100 mg/dL (5.6 mmol/L) to < 126 mg/dL (7.0 mmol/L), or a glycated hemoglobin (HbA1c) level of 5.7% to < 6.5%. Diabetes was defined by meeting any of the following criteria: a self-reported diagnosis of diabetes in the questionnaire, self-reported current use of insulin or glucose-lowering medications, an FPG level ≥ 126 mg/dL (7.0 mmol/L), or an HbA1c level ≥ 6.5%. Finally, normal glucose was defined as having an FPG < 100 mg/dL (5.6 mmol/L) and an HbA1c < 5.7%, with no self-reported history of diabetes or prediabetes. For missing data, multiple imputation using chained equations was performed. Definitions of other covariates and imputation methods for missing data are provided in Supplementary Material_1.

### Statistical analysis

Baseline characteristics of the study participants were compared using the Mann-Whitney U test for continuous variables and the chi-square or Fisher’s exact test for categorical variables. Cox proportional hazards models were used to estimate the hazard ratios (HRs) and 95% confidence intervals (CIs) for the association between HRT and all-cause and cardiovascular mortality. The proportional hazards assumption (PHA) was evaluated using Schoenfeld residuals. Because age violated the PHA, we employed an age-stratified Cox model (allowing for different baseline hazards across age strata) for all multivariable analyses. Our modeling strategy proceeded as follows: First, we assessed the main effect of HRT using crude and multivariable-adjusted models. Second, we tested for three-way interaction among HRT, EAA, and glycemic status in the fully adjusted model. Third, we performed glycemic-stratified analyses to examine the two-way interaction between HRT and EAA within each stratum. Sensitivity analyses included: (1) excluding participants with follow-up < 2 years to minimize reverse causation bias; (2) analyzing specific HRT regimens (estrogen-only versus combined estrogen-progestin therapy); and (3) conducting subgroup analyses by age categories (≤ 65 years and > 65 years) to evaluate potential effect modification. Statistical significance was set at two-sided *P* < 0.05. All analyses were performed using R software (version 4.4.1).

## Results

### Genetic association analysis of estrogen receptors and EAA

**Fig. 3:**
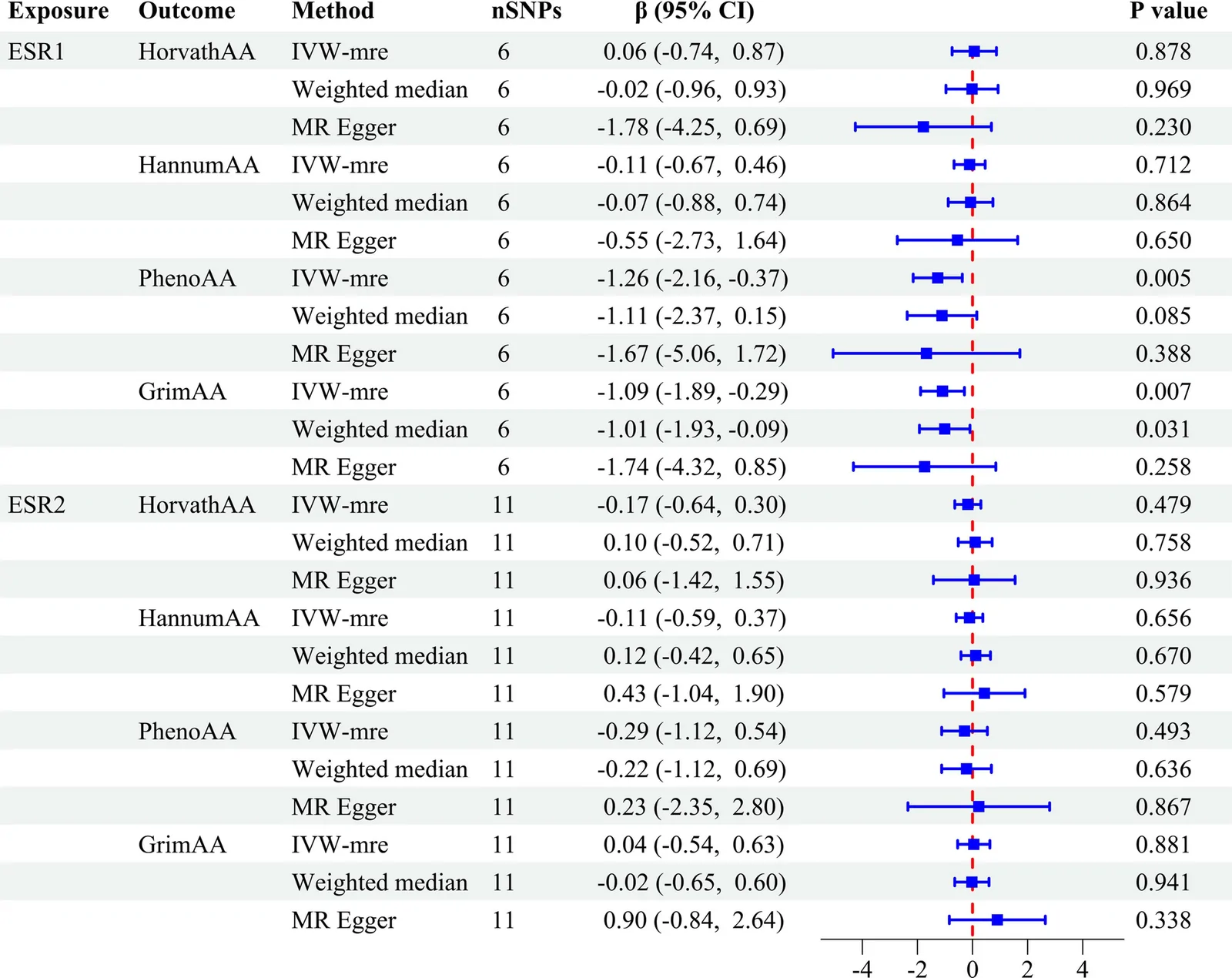
Forest plot of Mendelian randomization analysis for causal effects of estrogen receptor gene (ESR1/ESR2) expression on EAA phenotypes

Our MR analysis, using 6 and 11 genetic instruments for ESR1 and ESR2 respectively **(Table ****S2****)**, identified a significant protective effect of higher ESR1 expression on PhenoAA. Specifically, each 1-SD increase in genetically predicted ESR1 expression was causally associated with a 1.26-SD reduction in PhenoAA (β =−1.26; 95% CI,−2.16 to−0.37; Fig. 3). Conversely, no evidence of an association was found for ESR2 expression with any EAA biomarker, suggesting that the epigenetic aging effects of estrogen-based therapies may be mediated primarily via the ESR1 pathway. The robustness of these findings was confirmed through multiple sensitivity analyses. Details are available in Tables S3-S9 and Figures S1-S4.

### Participant characteristics from NHANES

To further explore the effects of HRT on epigenetic aging in postmenopausal women, we analyzed the NHANES cohort. The study included a total of 673 postmenopausal women, of whom 206 (30.6%) received HRT. Baseline characteristics of participants are detailed in Table 1. Compared with the non-HRT group, the HRT group was significantly younger and had lower PhenoAA, GrimAA, and Grim2AA (all *P* < 0.05). There was no significant difference in the age at menopause between the HRT and non-HRT groups, whereas a higher proportion of patients in the non-HRT group underwent hysterectomy. Demographically, the HRT group had a higher proportion of non-Hispanic whites and higher levels of education and income. Clinically, the HRT group showed lower prevalence of diabetes, hypertension, chronic kidney disease and obesity. With respect to lifestyle factors, the HRT group had higher rates of former smoking and alcohol consumption.

**Table 1:** Baseline characteristics of the study population by HRT in NHANES 1999–2002

Variable	HRT group (*n* = 206)	Non-HRT group (*n* = 467)	*P* value
Age, median (Q1–Q3)			
Chronological Age	61.00 (55.00, 66.00)	64.00 (59.00, 70.00)	< 0.001
< 65	137 (66.5%)	240 (51.4%)	
≥ 65	69 (33.5%)	227 (48.6%)	
HorvathAA	-0.34 (-3.16, 2.62)	-0.39 (-2.94, 2.98)	0.533
HannumAA	-0.37 (-2.93, 2.56)	-0.10 (-2.98, 3.03)	0.562
PhenoAA	-1.19 (-4.89, 2.61)	0.03 (-3.80, 4.80)	0.044
GrimAA	-1.57 (-3.71, 1.97)	-0.57 (-2.52, 2.28)	0.011
Grim2AA	-1.75 (-4.30, 1.75)	-0.24 (-2.87, 3.04)	< 0.001
Follow-up Time (month)	218 (205.00, 230.75)	211 (149.00, 231.00)	< 0.001
Menopause Age	45.00 (39.25, 50.00)	47.00 (39.00, 51.00)	0.258
Undergo Hysterectomy			< 0.001
Yes	194 (41.5%)	139 (67.5%)	
No	273 (58.5%)	67 (32.5%)	
Race			< 0.001
Non-Hispanic White	113 (54.9%)	129 (27.6%)	
Non-Hispanic Black	33 (16.0%)	128 (27.4%)	
Mexican American	49 (23.8%)	146 (31.3%)	
Hispanic and Other	11 (5.3%)	64 (13.7%)	
Education			< 0.001
Less than High School	48 (23.3%)	258 (55.2%)	
High School	59 (28.6%)	93 (19.9%)	
More than High School	99 (48.1%)	116 (24.8%)	
BMI (kg/m^2^)			< 0.001
< 25	79 (38.3%)	100 (21.4%)	
25 ~ 30	72 (35.0%)	126 (27.0%)	
> 30	55 (26.7%)	241 (51.6%)	
PIR			< 0.001
< 1.3	30 (14.6%)	184 (39.4%)	
1.3 ~ 3.5	82 (39.8%)	167 (35.8%)	
> 3.5	94 (45.6%)	116 (24.8%)	
Glycemic Status			< 0.001
Abnormal	82 (39.8%)	287 (61.5%)	
Normal	124 (60.2%)	180 (38.5%)	
Hypertension			< 0.001
Yes	140 (68.0%)	378 (80.9%)	
No	66 (32.0%)	89 (19.1%)	
Hyperlipidemia			0.167
Yes	175 (85.0%)	416 (89.1%)	
No	31 (15.0%)	51 (10.9%)	
Cardiovascular Diseases			0.602
Yes	27 (13.1%)	70 (15.0%)	
No	179 (86.9%)	397 (85.0%)	
Chronic Kidney Disease			0.003
Yes	32 (15.5%)	124 (26.6%)	
No	174 (84.5%)	343 (73.4%)	
Cancer			0.226
Yes	31 (15.0%)	53 (11.3%)	
No	175 (85.0%)	414 (88.7%)	
Smoking			0.012
Never	103 (50.0%)	281 (60.2%)	
Before	75 (36.4%)	118 (25.3%)	
Now	28 (13.6%)	68 (14.6%)	
Drinking			< 0.001
Yes	119 (57.8%)	183 (39.2%)	
No	87 (42.2%)	284 (60.8%)	

HRT, hormone replacement therapy; BMI, body mass index; PIR, poverty-to-income ratio

### Mortality risk assessment by HRT

In the fully adjusted model, HRT was associated with lower all-cause (HR 0.65; *P* = 0.006) and cardiovascular mortality (HR, 0.52; *P* = 0.033) **(**Table 2**)**. These associations persisted after excluding participants with less than 2 years of follow-up (Table S10). When stratified by treatment regimen, estrogen-only therapy was also associated with lower all-cause (HR, 0.64; *P* = 0.010) and cardiovascular mortality (HR, 0.44; *P* = 0.022) (Table S11); however, combined estrogen-progestin therapy showed no significant association (Table S12). When stratified by age, no significant associations were found between HRT and mortality (Tables S13-S14).

**Table 2:** Association of HRT with HRs for all-cause and cardiovascular mortality

	HR	95% CI	*P* value	PHA *P* value
All-Cause Mortality				
Model 1	0.54	(0.41, 0.72)	< 0.001	0.113
Model 2	0.61	(0.45, 0.82)	0.001	0.416
Model 3	0.65	(0.48, 0.89)	0.007	0.277
Model 4	0.65	(0.47, 0.88)	0.006	0.466
Cardiovascular Mortality				
Model 1	0.43	(0.25, 0.75)	0.003	0.058
Model 2	0.47	(0.27, 0.84)	0.011	0.294
Model 3	0.56	(0.31, 1.02)	0.059	0.224
Model 4	0.52	(0.28, 0.95)	0.033	0.504

Model 1: unadjusted for any covariates;

Model 2: stratified by age, and adjusted for race;

Model 3: stratified by age, and adjusted for race, education level, PIR, BMI, smoking status, and alcohol consumption;

Model 4: stratified by age, and adjusted for race, education level, PIR, BMI, smoking status, alcohol consumption, diabetes status, hypertension, hyperlipidemia, cardiovascular disease, chronic kidney disease, and cancer. HRT, hormone replacement therapy; HR, hazard ratio; CI, confidence interval; PHA, proportional hazards assumption; PIR, poverty-to-income ratio; BMI, body mass index

### Three-way interaction and glycemic-stratified two-way interaction analyses on mortality risk

To investigate whether glycemic status modified the joint effect of HRT and EAA on the risk of all-cause mortality, we conducted a three-way interaction analysis. The results indicated that abnormal glucose significantly altered the interactions of HRT with HorvathAA (HR = 1.14, *P* = 0.045) and PhenoAA (HR = 1.11, *P* = 0.023) on all-cause mortality. Further stratified analyses revealed that in the normal glucose subgroup, no significant two-way interactions were observed between HRT and any EAA indices (all P for interaction > 0.05). However, in the abnormal glucose subgroup, although the main effect of HRT exhibited a significant protective effect (HorvathAA: HR = 0.56, *P* = 0.013; PhenoAA: HR = 0.57, *P* = 0.014), HRT demonstrated a significant antagonistic interaction with these two EAA indices (HorvathAA interaction: HR = 1.11, *P* = 0.022; PhenoAA interaction: HR = 1.07, *P* = 0.028). Given that the protective main effect of HRT (HR < 1) and the interaction effect (HR > 1) were in opposite directions, this finding suggests that for patients with abnormal glucose, the survival benefit conferred by HRT is significantly attenuated as the degree of their epigenetic aging accelerates **(**Table 3**)**.

**Table 3:** Three-way interaction among HRT, EAA and glycemic status, along with glycemic-stratified two-way interactions on all-cause mortality risk

Variable	HorvathAA		HannumAA		PhenoAA		GrimAA		Grim2AA	
Three-way interaction/HR (95% CI), P value										
HRT × EAA × Abnormal Glucose	1.14 (1.00-1.30)	0.045	1.11 (0.96–1.27)	0.151	1.11 (1.01–1.22)	0.023	1.10 (0.97–1.25)	0.137	1.08 (0.96–1.21)	0.194
PHA P value	0.407		0.316		0.433		0.606		0.566	
Two-way interaction stratified by glycemic status/ HR (95% CI), P value										
Normal Glucose										
HRT	0.79 (0.47–1.32)	0.375	0.75 (0.45–1.26)	0.278	0.81 (0.48–1.35)	0.411	0.80 (0.48–1.35)	0.405	0.79 (0.47–1.32)	0.366
EAA	1.05 (1.01–1.10)	0.023	1.06 (1.01–1.10)	0.010	1.07 (1.03–1.12)	< 0.001	1.14 (1.07–1.21)	< 0.001	1.13 (1.07–1.20)	< 0.001
HRT × EAA	0.95 (0.87–1.05)	0.307	0.95 (0.87–1.05)	0.311	0.95 (0.88–1.02)	0.180	0.92 (0.84–1.01)	0.095	0.93 (0.86–1.02)	0.115
PHA P value	0.011		0.020		0.011		0.013		0.012	
Abnormal Glucose										
HRT	0.56 (0.36–0.89)	0.013	0.55 (0.35–0.87)	0.011	0.57 (0.36–0.89)	0.014	0.57 (0.35–0.92)	0.022	0.59 (0.36–0.95)	0.029
EAA	1.00 (0.97–1.03)	0.815	1.01 (0.98–1.03)	0.705	1.00 (0.98–1.02)	0.841	1.05 (1.01–1.10)	0.018	1.06 (1.02–1.10)	0.007
HRT × EAA	1.11 (1.02–1.22)	0.022	1.07 (0.96–1.19)	0.201	1.07 (1.01–1.14)	0.028	1.03 (0.94–1.13)	0.492	1.02 (0.94–1.11)	0.618
PHA P value	0.513		0.647		0.624		0.828		0.810	

All models were stratified by age and adjusted for race, education level, PIR, BMI, smoking status, alcohol consumption, hypertension, hyperlipidemia, cardiovascular disease, chronic kidney disease, and cancer. The three-way interaction terms (HRT × EAA × glycemic status) represent how the relationship between HRT and mortality risk is modified jointly by EAA and glycemic status. HRT, hormone replacement therapy; EAA, epigenetic age acceleration; HR, hazard ratio; CI, confidence interval; PHA, proportional hazards assumption; PIR, poverty-to-income ratio; BMI, body mass index

These findings demonstrated a degree of robustness and specificity across a series of sensitivity analyses. After excluding participants with a follow-up duration of less than 2 years, the three-way interactions for HorvathAA and PhenoAA remained significant, although the two-way interaction between HRT and PhenoAA was somewhat attenuated (Table S15). Furthermore, this complex interaction pattern appeared to be related to the specific treatment regimen: the aforementioned interactions persisted in the subgroup receiving estrogen-alone therapy (Table S16), whereas no significant interactions were observed in the combined estrogen-progestin therapy subgroup (Table S17). Finally, no significant interactions were detected in the age-stratified analyses for all-cause mortality (Tables S18-S19) or in any of the evaluations assessing cardiovascular mortality (Tables S20-S25).

## Discussion

Through MR and the NHANES cohort analysis, this study found that elevated ESR1 expression levels can decelerate PhenoAA, and HRT is significantly associated with decreased risks of all-cause and cardiovascular mortality. A three-way interaction analysis revealed that abnormal glucose significantly modified the joint effect of HRT and two types of EAA (HorvathAA and PhenoAA) on all-cause mortality. Stratified analyses showed no significant interaction in the normal glucose group, but a significant antagonistic interaction between HRT and two types of EAA (HorvathAA and PhenoAA) in the abnormal glucose group. Consequently, for patients with abnormal glucose, the survival benefits conferred by HRT are significantly attenuated as their epigenetic aging accelerates.

The distinct roles of the two estrogen receptor subtypes in aging can be attributed to their differing epigenetic and physiological regulation. At the molecular level, ESR1 exhibits a dual regulatory pattern, where methylation at its promoter is linked to age-related expression decline, while methylation at distal sites responds to aging or estrogen deprivation, allowing for dynamic responsiveness to the aging process [31]. In contrast, epigenetic regulation of ESR2, primarily via promoter methylation, is more closely associated with tissue-specific expression than with age-related changes [32]. These differences are mirrored at the physiological level. For instance, in estrogen receptor knockout models, ESR1’s compensatory transcriptional enhancement capacity declines with advancing age, whereas ESR2 lacks such feedback regulatory properties [33]. Furthermore, intervention studies show that estrogen treatment can specifically upregulate ESR2 in localized regions like the hippocampus but has limited effects on ESR1, suggesting ESR2 is more involved in localized adaptive responses [34, 35]. Therefore, based on the results of our MR analysis, we further speculate that ESR1 plays a dominant role in mediating the extensive regulation of the overall aging process by estrogen. In contrast, the effects of ESR2 may be more limited, or dependent on specific physiological contexts and tissue microenvironments, and its impact on the global epigenetic clock may not be significant.

This study highlights the central role of glycemic status in modulating the combined effects of HRT and EAA. We found that the significant survival benefits of HRT were almost exclusively confined to individuals with abnormal glucose; however, this protective effect was substantially attenuated as HorvathAA and PhenoAA increased, as evidenced by significant bidirectional and three-way interaction effects. The underlying basis for this differential interaction lies in the fundamentally distinct construction logic of different epigenetic clocks. As a first-generation clock, HorvathAge is essentially a “timekeeper” reflecting intrinsic cellular aging and stem cell exhaustion [5]. Although exogenous estrogen, acting via ESR1, exerts anti-inflammatory, metabolic-improving, and mitochondrial-protective effects [36, 37], when a patient’s HorvathAA is extremely elevated—indicating that profound, irreversible methylation drift has already occurred at the cellular level—the capacity of target tissues to respond to estrogenic signaling may be severely blunted. In other words, severe intrinsic cellular aging constitutes a “ceiling effect” that limits the protective efficacy of HRT. Correspondingly, the PhenoAge model was constructed by directly integrating multiple clinical biomarkers closely associated with chronic inflammation and metabolic dysregulation, including glucose, albumin, creatinine, and C-reactive protein [5]. In the context of abnormal glucose, elevated PhenoAA reflects a state in which patients are already burdened with superimposed systemic inflammation and severe metabolic derangement. Under such an extreme phenotype of multi-system functional decline, HRT alone may be insufficient to reverse the complex pathological processes, thereby manifesting as a significant attenuation of the protective effect of HRT. In stark contrast, HannumAge is a first-generation clock trained on whole-blood data with a greater emphasis on immune system aging, such as shifts in immune cell proportions [38]. As such, it is less sensitive to endocrine and glucose metabolic interactions than PhenoAge. On the other hand, although GrimAge and its updated iteration (Grim2Age) demonstrated the strongest independent predictive power for all-cause mortality in both groups, no significant interaction with HRT was observed. This is because GrimAge is a “second-generation clock” trained directly on downstream terminal indicators associated with mortality—including plasma proteins such as plasminogen activator inhibitor−1 and growth differentiation factor−15, as well as smoking exposure—and thus functions primarily as an ultimate marker of absolute lifespan attrition [39–41]. When disease progression has reached the terminal-stage risk captured by GrimAge, its powerful prognostic weight may overshadow the potential effect modification by HRT. Therefore, our findings suggest that HorvathAge and PhenoAge may be better suited as predictive biomarkers for clinically evaluating the potential benefits of HRT.

On the one hand, multiple epigenetic studies have confirmed that abnormal glucose is closely associated with accelerated biological aging. Wu et al. demonstrated that in populations with diabetes and prediabetes, DNA methylation age acceleration not only significantly increases the risks of all-cause and cardiovascular mortality but also plays a crucial mediating role between various health-related exposures and all-cause mortality [17]. Shemer et al. also confirmed that type 2 diabetes (T2D) is significantly associated with accelerated biological aging, further increasing susceptibility to other age-related diseases [42]. Similarly, Sabbatinelli et al. found that epigenetic clocks can effectively predict mortality risk in patients with T2D, independent of traditional risk factors [43]. Essentially, EAA reflects the cumulative effects of cellular damage, chronic inflammation, and metabolic disorders, which are deeply intertwined with the pathophysiological processes of diabetes. On the other hand, the metabolic benefits of HRT are widely recognized. Kim et al. demonstrated that HRT is inversely correlated with the prevalence of metabolic syndrome and can effectively reduce key metabolic parameters in women, including fasting blood glucose, total cholesterol, and systolic blood pressure [44]. Complementing these findings, Paschou et al. further highlighted that HRT improves glucose homeostasis, reduces the risk of new-onset T2D, and optimizes glycemic control in women with established T2D [45]. Although the aforementioned mechanisms suggest that HRT holds the potential to delay aging through metabolic improvements, our findings indicate that these limited benefits may be insufficient to counteract the profound burden of aging acceleration already established in patients with abnormal glucose. In this context, the protective effect of standalone HRT is inevitably and significantly attenuated.

Several key limitations of this study warrant acknowledgment. First, the MR analyses employed a cross-ancestry design (European and African populations), which may introduce bias in effect estimates due to differences in linkage disequilibrium structure and allele frequencies. However, according to a recent methodological study by Li and Morrison [46], ancestry mismatch typically results in attenuation bias—that is, effect estimates are shifted toward the null—and rarely gives rise to false positives or misidentification of causal direction. Accordingly, the significant retarding effect of ESR1 expression on PhenoAA observed in this study is more likely a conservative underestimate than a spurious association, though the precise magnitude of the effect should still be interpreted with caution. Second, selection bias may have been introduced because our analysis was restricted to postmenopausal participants with complete biological, clinical, and covariate data. We therefore compared the included participants with excluded postmenopausal NHANES participants and found significant differences in demographic, socioeconomic, and health-related characteristics (Table S26). The included sample was not healthier or wealthier; instead, it included higher proportions of ethnic minority participants, individuals with lower education and income, and participants with abnormal glycemic status, hypertension, hyperlipidemia, cardiovascular disease, chronic kidney disease, and obesity. Therefore, the possibility of selection bias cannot be fully ruled out, and the generalizability of our findings should be interpreted cautiously. Nevertheless, the direction of this bias does not suggest a typical healthy-volunteer effect. Rather, our findings may be especially applicable to socioeconomically disadvantaged and clinically vulnerable postmenopausal women, but further validation in broader populations is needed. Third, the definition of HRT exposure in this study was necessarily crude, constrained by data availability. Medication information was based solely on self-reported use within the past month, lacking critical details such as initiation date, treatment duration, dosage, and route of administration. This broad classification may have introduced misclassification bias. Fourth, we did not perform multiple testing correction for the observational analyses, which may increase the risk of false-positive results. Therefore, these findings, particularly the interaction analyses, should be considered exploratory. Fifth, this study relied on DNAm data derived from peripheral blood, which may not accurately capture the tissue-specific epigenetic effects of HRT in key target organs. Notwithstanding these limitations, this study provides novel preliminary evidence regarding the complex interplay among estrogen signaling, epigenetic aging, and glycemic status. Future investigations should prioritize prospective longitudinal designs, with refined characterization of the effects of treatment timing, duration, and formulation, as well as in-depth examination of tissue-specific epigenetic responses, in order to fully elucidate the mechanisms underlying HRT in the aging process.

## Conclusions

In conclusion, estrogens may decelerate phenotypic epigenetic aging (PhenoAA) via the ESR1 pathway. In postmenopausal women, HRT—particularly estrogen-only regimens—is significantly associated with reduced all-cause and cardiovascular mortality. However, this survival benefit is significantly modified by glycemic status; in patients with abnormal glucose, accelerated HorvathAA or PhenoAA antagonizes and substantially attenuates the protective effects of HRT. Future studies should evaluate whether incorporating EAA and glycemic status into HRT decision-making can optimize clinical outcomes.

## Supplementary Information

Below is the link to the electronic supplementary material.

Supplementary Material 1

Supplementary Material 2

## Data Availability

All data used in this study are publicly available. Summary statistics of eQTLs associated with ESR1 and ESR2 gene expression can be obtained from the eQTLGen consortium (https://eqtlgen.org/). GWAS summary statistics for EAA can be obtained from the GWAS Catalog (https://www.ebi.ac.uk/gwas/publications/34187551). The NHANES-related data demonstrated in this study can be found on the NHANES website (https://www.cdc.gov/nchs/nhanes/).

## Abbreviations

CI: Confidence interval
DNAm: DNA methylation
EAA: Epigenetic age acceleration
eQTL: Expression quantitative trait loci
ESR1: Estrogen receptorα
ESR2: Estrogen receptorβ
GWAS: Genome-wide association study
HR: Hazard ratio
HRT: Hormone replacement therapy
IV: Instrumental variable
IVW-mre: Multiplicative-random-effects inverse variance weighted
MR: Mendelian randomization
MR-PRESSO: MR pleiotropy residual sum and outlier
NHANES: National Health and Nutrition Examination Survey
PHA: Proportional hazards assumption
SD: Standard deviation
SNP: Single nucleotide polymorphism
T2D: Type 2 Diabetes
