# Supplementary material for: Hormone replacement therapy through the lens of epigenetic clock: estrogen signals, glycemic status, and lifespan
Source: Biol Sex Differ. 2026 Jul 31;17:172. doi: 10.1186/s13293-026-00959-w (PMC13715200; doi:10.1186/s13293-026-00959-w)
Supplement: Supplementary file 1 [file 13293_2026_959_MOESM2_ESM.docx]

**Hormone Replacement Therapy Through the Lens of Epigenetic Clock:**

**Estrogen Signals, Glycemic Status, and Lifespan**

**Appendix 1. Variable definition**

Age was categorized as <65 and ≥65 years. Race was categorized into non-Hispanic White, non-Hispanic Black, Mexican American, and Hispanic and other races. Educational level was categorized into below high school, high school, and above high school. Poverty income ratio (PIR) was categorized into three levels: low (< 1.3), medium (1.3-3.5), and high (> 3.5). Body mass index (BMI, kg/m²) was categorized into normal (< 25), overweight (25-30), and obese (≥ 30). Smoking status was categorized into current smokers (those who had smoked at least 100 cigarettes in their lifetime and currently smoke), former smokers (those who had smoked at least 100 cigarettes in their lifetime but do not currently smoke), and never smokers (those who had not smoked 100 cigarettes in their lifetime). Alcohol consumption was categorized into drinkers (those who had consumed at least 12 drinks in the past year) and non-drinkers (those who had consumed fewer than 12 drinks in the past year or had never consumed alcohol). Hypertension was defined as self-reported physician diagnosis of hypertension in the questionnaire, current use of antihypertensive medications, previous receipt of treatment recommendations for hypertension, mean systolic blood pressure ≥ 130 mmHg, or mean diastolic blood pressure ≥ 80 mmHg, with meeting any one of these criteria being sufficient for classification as hypertension. Hyperlipidemia was defined as physician diagnosis of high cholesterol, current use of lipid-lowering medications, total cholesterol ≥ 200 mg/dL, high-density lipoprotein (HDL) < 40 mg/dL for men (< 50 mg/dL for women), triglycerides (TG) ≥ 150 mg/dL, or low-density lipoprotein (LDL) ≥ 130 mg/dL, with meeting any one of these criteria being sufficient for classification as hyperlipidemia. Cardiovascular disease was defined as a history of congestive heart failure, previous diagnosis of coronary heart disease, previous diagnosis of angina, previous heart attack, or previous diagnosis of stroke, with meeting any one of these criteria being sufficient for classification as having a history of cardiovascular disease. Chronic kidney disease was defined as physician-confirmed diagnosis of chronic kidney disease, urinary albumin-to-creatinine ratio ≥ 30 mg/g, or estimated glomerular filtration rate (eGFR) < 60 mL/min/1.73m², with meeting any one of these criteria being sufficient for classification as chronic kidney disease. Cancer was defined as having been previously diagnosed with cancer by a physician. More detailed information about covariates can be obtained at http://www.cdc.gov/nchs/nhanes/.

**Appendix 2. Missing Data and Imputation Methods**

Missing data rates for the main study variables were as follows: PIR 10.70%, BMI 2.23%, smoking status 0.15%, alcohol consumption 1.04%, glycemic status 0.30%, hypertension 0.15%, hyperlipidemia 0.89%, cardiovascular disease 2.23%, chronic kidney disease 0.45%, and cancer 0.15%. Except for the PIR, the missing rates of the remaining nine variables were all below 5%, indicating good data quality in this study. The relatively high missing rate of the PIR is consistent with typical patterns in socioeconomic surveys, primarily related to participants' sensitivity regarding income information disclosure. For missing values, this study employed multiple imputation using chained equations for processing, which was specifically implemented using the 'mice' package in R software. Based on the characteristics of variable types, we applied multinomial logistic regression models for multinomial categorical variables and binary logistic regression models for binary variables during imputation. For parameter settings, we generated 10 complete imputed datasets, set the maximum number of iterations to 20, and used a prediction matrix with a minimum correlation coefficient threshold of 0.1 to screen potential predictor variables. To facilitate subsequent statistical analysis, we ultimately used the mode method to integrate the multiple imputation results into a single complete dataset, ensuring that the variable structure remained consistent with the original data.

**Appendix 3. Death Outcome**

|  | HRT group  (n=206) | Non-HRT group  (n=467) | Normal Glucose (n=304) | Abnormal Glucose (n=369) |
| --- | --- | --- | --- | --- |
| Survival | 146 (70.9%) | 250 (53.5%) | 201 (66.1%) | 195 (52.8%) |
| Death | 60 (29.1%) | 217 (46.5%) | 103 (33.9%) | 174 (47.2%) |
| Cardiovascular death | 15 (7.3%) | 69 (14.8%) | 32 (10.5%) | 52 (14.1%) |

**Contents**

**Table S10.** Sensitivity analysis: association of HRT with HRs for all-cause and cardiovascular mortality after excluding participants with less than 2 years of follow-up.

**Table S11.** Sensitivity analysis: association of estrogen-only therapy with HRs for all-cause and cardiovascular mortality.

**Table S12.** Sensitivity analysis: association of estrogen/progestin combined therapy with HRs for all-cause and cardiovascular mortality.

**Table S13.** Sensitivity analysis: association of HRT with HRs for all-cause and cardiovascular mortality among participants aged 65 years or older.

**Table S14.** Sensitivity analysis: association of HRT with HRs for all-cause and cardiovascular mortality among participants aged younger than 65 years.

**Table S15.** Sensitivity analysis: three-way interaction of HRT, EAA and glycemic status, along with glycemic-stratified two-way interaction analysis on all-cause mortality risk excluding participants with less than 2 years of follow-up.

**Table S16.** Sensitivity analysis: three-way interaction of estrogen-only therapy, EAA and glycemic status, along with glycemic-stratified two-way interaction analysis on all-cause mortality risk.

**Table S17.** Sensitivity analysis: three-way interaction of estrogen-progestin combined therapy, EAA and glycemic status, along with glycemic-stratified two-way interaction analysis on all-cause mortality risk.

**Table S18.** Sensitivity analysis: three-way interaction of HRT, EAA and glycemic status, along with glycemic-stratified two-way interaction analysis on all-cause mortality risk among participants aged 65 years or older.

**Table S19.** Sensitivity analysis: three-way interaction of HRT, EAA and glycemic status, along with glycemic-stratified two-way interaction analysis on all-cause mortality risk among participants aged younger than 65 years.

**Table S20.** Three-way interaction of HRT, EAA and glycemic status, along with glycemic-stratified two-way interaction analysis on cardiovascular mortality risk.

**Table S21.** Sensitivity analysis: three-way interaction of HRT, EAA and glycemic status, along with glycemic-stratified two-way interaction analysis on cardiovascular mortality risk excluding participants with less than 2 years of follow-up.

**Table S22.** Sensitivity analysis: three-way interaction of estrogen-only therapy, EAA and glycemic status, along with glycemic-stratified two-way interaction analysis on cardiovascular mortality risk.

**Table S23.** Sensitivity analysis: three-way interaction of estrogen-progestin combined therapy, EAA and glycemic status, along with glycemic-stratified two-way interaction analysis on cardiovascular mortality risk.

**Table S24.** Sensitivity analysis: three-way interaction of HRT, EAA and glycemic status, along with glycemic-stratified two-way interaction analysis on cardiovascular mortality risk among participants aged 65 years or older.

**Table S25.** Sensitivity analysis: three-way interaction of HRT, EAA and glycemic status, along with glycemic-stratified two-way interaction analysis on cardiovascular mortality risk among participants aged younger than 65 years.

**Table S26.** Comparison of baseline characteristics between included and excluded postmenopausal women.

**Figure S1.** The leave-one-out plot of Mendelian randomization analysis results.

**Figure S2.** The forest plot of Mendelian randomization analysis results.

**Figure S3.** The funnel plot of Mendelian randomization analysis results.

**Figure S4.** The scatter plot of Mendelian randomization analysis results.

**Table S10.** Sensitivity analysis: association of HRT with HRs for all-cause and cardiovascular mortality after excluding participants with less than 2 years of follow-up.

|  | HR | 95% CI | P value | PHA P value |
| --- | --- | --- | --- | --- |
| All-Cause Mortality | | | | |
| Model 1 | 0.53 | (0.40, 0.71) | <0.001 | 0.051 |
| Model 2 | 0.60 | (0.44, 0.81) | <0.001 | 0.204 |
| Model 3 | 0.64 | (0.47, 0.88) | 0.006 | 0.197 |
| Model 4 | 0.62 | (0.45, 0.86) | 0.004 | 0.262 |
| Cardiovascular Mortality | | | | |
| Model 1 | 0.45 | (0.26, 0.79) | 0.006 | 0.096 |
| Model 2 | 0.50 | (0.28, 0.89) | 0.019 | 0.390 |
| Model 3 | 0.58 | (0.32, 1.07) | 0.082 | 0.184 |
| Model 4 | 0.54 | (0.29, 0.99) | 0.047 | 0.361 |

Model 1: unadjusted for any covariates;
Model 2: stratified by age, and adjusted for race;
Model 3: stratified by age, and adjusted for race, education level, PIR, BMI, smoking status, and alcohol consumption;
Model 4: stratified by age, and adjusted for race, education level, PIR, BMI, smoking status, alcohol consumption, diabetes status, hypertension, hyperlipidemia, cardiovascular disease, chronic kidney disease, and cancer. HRT, hormone replacement therapy; HR, hazard ratio; CI, confidence interval; PHA, proportional hazards assumption; PIR, poverty-to-income ratio; BMI, body mass index.

**Table S11.** Sensitivity analysis: association of estrogen-only therapy with HRs for all-cause and cardiovascular mortality.

|  | HR | 95% CI | P value | PHA P value |
| --- | --- | --- | --- | --- |
| All-Cause Mortality | | | | |
| Model 1 | 0.63 | (0.46, 0.86) | 0.004 | 0.125 |
| Model 2 | 0.66 | (0.48, 0.92) | 0.013 | 0.372 |
| Model 3 | 0.69 | (0.49, 0.96) | 0.028 | 0.389 |
| Model 4 | 0.64 | (0.46, 0.90) | 0.010 | 0.632 |
| Cardiovascular Mortality | | | | |
| Model 1 | 0.42 | (0.22, 0.82) | 0.011 | 0.287 |
| Model 2 | 0.44 | (0.23, 0.87) | 0.019 | 0.391 |
| Model 3 | 0.50 | (0.25, 1.01) | 0.052 | 0.325 |
| Model 4 | 0.44 | (0.22, 0.89) | 0.022 | 0.703 |

Model 1: unadjusted for any covariates;
Model 2: stratified by age, and adjusted for race;
Model 3: stratified by age, and adjusted for race, education level, PIR, BMI, smoking status, and alcohol consumption;
Model 4: stratified by age, and adjusted for race, education level, PIR, BMI, smoking status, alcohol consumption, diabetes status, hypertension, hyperlipidemia, cardiovascular disease, chronic kidney disease, and cancer. HRT, hormone replacement therapy; HR, hazard ratio; CI, confidence interval; PHA, proportional hazards assumption; PIR, poverty-to-income ratio; BMI, body mass index.

**Table S12.** Sensitivity analysis: association of estrogen/progestin combined therapy with HRs for all-cause and cardiovascular mortality.

|  | HR | 95% CI | P value | PHA P value |
| --- | --- | --- | --- | --- |
| All-Cause Mortality | | | | |
| Model 1 | 0.36 | (0.21, 0.64) | <0.001 | 0.471 |
| Model 2 | 0.49 | (0.28, 0.87) | 0.014 | 0.692 |
| Model 3 | 0.56 | (0.31, 1.02) | 0.056 | 0.482 |
| Model 4 | 0.67 | (0.37, 1.22) | 0.192 | 0.651 |
| Cardiovascular Mortality | | | | |
| Model 1 | 0.44 | (0.18, 1.10) | 0.080 | 0.050 |
| Model 2 | 0.62 | (0.24, 1.56) | 0.308 | 0.349 |
| Model 3 | 0.72 | (0.27, 1.93) | 0.517 | 0.278 |
| Model 4 | 0.84 | (0.31, 2.30) | 0.740 | 0.534 |

Model 1: unadjusted for any covariates;
Model 2: stratified by age, and adjusted for race;
Model 3: stratified by age, and adjusted for race, education level, PIR, BMI, smoking status, and alcohol consumption;
Model 4: stratified by age, and adjusted for race, education level, PIR, BMI, smoking status, alcohol consumption, diabetes status, hypertension, hyperlipidemia, cardiovascular disease, chronic kidney disease, and cancer. HRT, hormone replacement therapy; HR, hazard ratio; CI, confidence interval; PHA, proportional hazards assumption; PIR, poverty-to-income ratio; BMI, body mass index.

**Table S13.** Sensitivity analysis: association of HRT with HRs for all-cause and cardiovascular mortality among participants aged 65 years or older.

|  | HR | 95% CI | P value | PHA P value |
| --- | --- | --- | --- | --- |
| All-Cause Mortality | | | | |
| Model 1 | 0.73 | (0.51, 1.06) | 0.098 | 0.326 |
| Model 2 | 0.67 | (0.46, 0.98) | 0.039 | 0.637 |
| Model 3 | 0.68 | (0.45, 1.01) | 0.053 | 0.732 |
| Model 4 | 0.69 | (0.46, 1.02) | 0.065 | 0.962 |
| Cardiovascular Mortality | | | | |
| Model 1 | 0.52 | (0.24, 1.10) | 0.086 | 0.383 |
| Model 2 | 0.46 | (0.21, 1.01) | 0.052 | 0.360 |
| Model 3 | 0.49 | (0.22, 1.11) | 0.087 | 0.477 |
| Model 4 | 0.48 | (0.21, 1.08) | 0.076 | 0.595 |

Model 1: unadjusted for any covariates;
Model 2: stratified by age, and adjusted for race;
Model 3: stratified by age, and adjusted for race, education level, PIR, BMI, smoking status, and alcohol consumption;
Model 4: stratified by age, and adjusted for race, education level, PIR, BMI, smoking status, alcohol consumption, diabetes status, hypertension, hyperlipidemia, cardiovascular disease, chronic kidney disease, and cancer. HRT, hormone replacement therapy; HR, hazard ratio; CI, confidence interval; PHA, proportional hazards assumption; PIR, poverty-to-income ratio; BMI, body mass index.
**Table S14.** Sensitivity analysis: association of HRT with HRs for all-cause and cardiovascular mortality among participants aged younger than 65 years.

|  | HR | 95% CI | P value | PHA P value |
| --- | --- | --- | --- | --- |
| All-Cause Mortality | | | | |
| Model 1 | 0.51 | (0.32, 0.81) | 0.004 | 0.120 |
| Model 2 | 0.54 | (0.34, 0.87) | 0.011 | 0.624 |
| Model 3 | 0.64 | (0.39, 1.06) | 0.085 | 0.340 |
| Model 4 | 0.64 | (0.38, 1.08) | 0.095 | 0.052 |
| Cardiovascular Mortality | | | | |
| Model 1 | 0.46 | (0.20, 1.06) | 0.068 | 0.240 |
| Model 2 | 0.49 | (0.21, 1.15) | 0.102 | 0.776 |
| Model 3 | 0.77 | (0.30, 1.99) | 0.593 | 0.736 |
| Model 4 | 0.73 | (0.27, 1.96) | 0.532 | 0.758 |

Model 1: unadjusted for any covariates;
Model 2: stratified by age, and adjusted for race;
Model 3: stratified by age, and adjusted for race, education level, PIR, BMI, smoking status, and alcohol consumption;
Model 4: stratified by age, and adjusted for race, education level, PIR, BMI, smoking status, alcohol consumption, diabetes status, hypertension, hyperlipidemia, cardiovascular disease, chronic kidney disease, and cancer. HRT, hormone replacement therapy; HR, hazard ratio; CI, confidence interval; PHA, proportional hazards assumption; PIR, poverty-to-income ratio; BMI, body mass index.

**Table S15.** Sensitivity analysis: three-way interaction of HRT, EAA and glycemic status, along with glycemic-stratified two-way interaction analysis on all-cause mortality risk excluding participants with less than 2 years of follow-up.

| Variable | HorvathAA | | HannumAA | | PhenoAA | | GrimAA | | Grim2AA | |
| --- | --- | --- | --- | --- | --- | --- | --- | --- | --- | --- |
| Three-way interaction/HR (95% CI), P value | | | | | | | | | | |
| HRT × EAA ×  Abnormal Glucose | 1.15 (1.01-1.32) | 0.035 | 1.12 (0.97-1.29) | 0.117 | 1.10 (1.00-1.21) | 0.049 | 1.09 (0.96-1.24) | 0.179 | 1.07 (0.95-1.20) | 0.271 |
| PHA P value | 0.302 | | 0.231 | | 0.222 | | 0.442 | | 0.386 | |
| Two-way interaction stratified by glycemic status/ HR (95% CI), P value | | | | | | | | | | |
| Normal Glucose | | | | | | | | | | |
| HRT | 0.81 (0.48-1.36) | 0.424 | 0.77 (0.46-1.30) | 0.330 | 0.82 (0.48-1.39) | 0.463 | 0.81 (0.48-1.38) | 0.447 | 0.80 (0.47-1.36) | 0.411 |
| EAA | 1.05 (1.00-1.10) | 0.031 | 1.06 (1.01-1.10) | 0.011 | 1.08 (1.03-1.12) | <0.001 | 1.13 (1.06-1.21) | <0.001 | 1.13 (1.06-1.20) | <0.001 |
| HRT × EAA | 0.95 (0.86-1.04) | 0.274 | 0.95 (0.87-1.05) | 0.335 | 0.95 (0.88-1.03) | 0.237 | 0.92 (0.83-1.01) | 0.089 | 0.93 (0.85-1.02) | 0.114 |
| PHA P value | 0.003 | | 0.006 | | 0.003 | | 0.002 | | 0.002 | |
| Abnormal Glucose | | | | | | | | | | |
| HRT | 0.51 (0.32-082) | 0.006 | 0.50 (0.31-0.81) | 0.005 | 0.53 (0.33-0.84) | 0.007 | 0.53 (0.32-0.87) | 0.012 | 0.55 (0.34-0.89) | 0.016 |
| EAA | 1.01 (0.98-1.04) | 0.708 | 1.01 (0.98-1.04) | 0.650 | 1.00 (0.98-1.03) | 0.788 | 1.05 (1.00-1.10) | 0.042 | 1.05 (1.01-1.10) | 0.019 |
| HRT × EAA | 1.12 (1.02-1.24) | 0.018 | 1.08 (0.97-1.21) | 0.160 | 1.06 (1.00-1.13) | 0.061 | 1.03 (0.93-1.13) | 0.593 | 1.01 (0.93-1.10) | 0.780 |
| PHA P value | 0.362 | | 0.510 | | 0.300 | | 0.583 | | 0.536 | |

All models were stratified by age and adjusted for race, education level, PIR, BMI, smoking status, alcohol consumption, hypertension, hyperlipidemia, cardiovascular disease, chronic kidney disease, and cancer. The three-way interaction terms (HRT × EAA × glycemic status) represent how the relationship between HRT and mortality risk is modified jointly by EAA and glycemic status. HRT, hormone replacement therapy; EAA, epigenetic age acceleration; HR, hazard ratio; CI, confidence interval; PHA, proportional hazards assumption; PIR, poverty-to-income ratio; BMI, body mass index.

**Table S16.** Sensitivity analysis: three-way interaction of estrogen-only therapy, EAA and glycemic status, along with glycemic-stratified two-way interaction analysis on all-cause mortality risk.

| Variable | HorvathAA | | HannumAA | | PhenoAA | | GrimAA | | Grim2AA | |
| --- | --- | --- | --- | --- | --- | --- | --- | --- | --- | --- |
| Three-way interaction/HR (95% CI), P value | | | | | | | | | | |
| Estrogen-only × EAA × Abnormal Glucose | 1.20 (1.03-1.39) | 0.016 | 1.13 (0.98-1.32) | 0.100 | 1.11 (1.00-1.22) | 0.049 | 1.07 (0.93-1.23) | 0.333 | 1.05 (0.93-1.19) | 0.443 |
| PHA P value | 0.522 | | 0.477 | | 0.530 | | 0.828 | | 0.792 | |
| Two-way interaction stratified by glycemic status/ HR (95% CI), P value | | | | | | | | | | |
| Normal Glucose | | | | | | | | | | |
| Estrogen-only | 0.76 (0.43-1.33) | 0.332 | 0.75 (0.43-1.29) | 0.299 | 0.80 (0.46-1.39) | 0.433 | 0.78 (0.45-1.36) | 0.385 | 0.78 (0.45-1.35) | 0.379 |
| EAA | 1.05 (1.01-1.10) | 0.026 | 1.05 (1.01-1.10) | 0.013 | 1.07 (1.03-1.12) | <0.001 | 1.14 (1.07-1.21) | <0.001 | 1.13 (1.07-1.20) | <0.001 |
| Estrogen-only × EAA | 0.89 (0.80-1.01) | 0.064 | 0.94 (0.85-1.04) | 0.212 | 0.95 (0.88-1.03) | 0.231 | 0.95 (0.86-1.05) | 0.348 | 0.96 (0.88-1.05) | 0.388 |
| PHA P value | 0.053 | | 0.113 | | 0.063 | | 0.082 | | 0.082 | |
| Abnormal Glucose | | | | | | | | | | |
| Estrogen-only | 0.52 (0.32-0.86) | 0.012 | 0.53 (0.32-0.88) | 0.014 | 0.55 (0.34-0.90) | 0.018 | 0.56 (0.33-0.94) | 0.030 | 0.58 (0.34-0.97) | 0.039 |
| EAA | 1.00 (0.97-1.03) | 0.804 | 1.01 (0.98-1.04) | 0.652 | 1.00 (0.98-1.02) | 0.825 | 1.05 (1.01-1.10) | 0.020 | 1.06 (1.01-1.10) | 0.008 |
| Estrogen-only × EAA | 1.12 (1.01-1.24) | 0.031 | 1.07 (0.95-1.20) | 0.247 | 1.06 (0.99-1.13) | 0.091 | 1.02 (0.92-1.13) | 0.706 | 1.01 (0.92-1.10) | 0.863 |
| PHA P value | 0.352 | | 0.555 | | 0.299 | | 0.648 | | 0.611 | |

All models were stratified by age and adjusted for race, education level, PIR, BMI, smoking status, alcohol consumption, hypertension, hyperlipidemia, cardiovascular disease, chronic kidney disease, and cancer. The three-way interaction terms (Estrogen-only therapy × EAA × glycemic status) represent how the relationship between estrogen-only therapy and mortality risk is modified jointly by EAA and glycemic status. EAA, epigenetic age acceleration; HR, hazard ratio; CI, confidence interval; PHA, proportional hazards assumption; PIR, poverty-to-income ratio; BMI, body mass index.

**Table S17.** Sensitivity analysis: three-way interaction of estrogen-progestin combined therapy, EAA and glycemic status, along with glycemic-stratified two-way interaction analysis on all-cause mortality risk.

| Variable | HorvathAA | | HannumAA | | PhenoAA | | GrimAA | | Grim2AA | |
| --- | --- | --- | --- | --- | --- | --- | --- | --- | --- | --- |
| Three-way interaction/HR (95% CI), P value | | | | | | | | | | |
| Estrogen-progestin× EAA × Abnormal Glucose | 1.05 (0.79-1.40) | 0.745 | 1.00 (0.72-1.39) | 0.998 | 1.17 (0.95-1.45) | 0.148 | 1.23 (0.97-1.56) | 0.088 | 1.21 (0.97-1.52) | 0.095 |
| PHA P value | 0.570 | | 0.569 | | 0.395 | | 0.690 | | 0.658 | |
| Two-way interaction stratified by glycemic status/ HR (95% CI), P value | | | | | | | | | | |
| Normal Glucose | | | | | | | | | | |
| Estrogen-progestin | 0.59 (0.24-1.42) | 0.237 | 0.49 (0.20-1.23) | 0.129 | 0.54 (0.21-1.37) | 0.192 | 0.48 (0.18-1.26) | 0.137 | 0.44 (0.16-1.20) | 0.109 |
| EAA | 1.05 (1.00-1.10) | 0.038 | 1.05 (1.01-1.10) | 0.016 | 1.08 (1.03-1.12) | <0.001 | 1.15 (1.07-1.23) | <0.001 | 1.14 (1.07-1.22) | <0.001 |
| Estrogen-progestin× EAA | 1.12 (0.94-1.33) | 0.202 | 1.03 (0.83-1.27) | 0.812 | 0.95 (0.79-1.15) | 0.595 | 0.82 (0.66-1.02) | 0.079 | 0.84 (0.69-1.03) | 0.087 |
| PHA P value | 0.176 | | 0.153 | | 0.081 | | 0.236 | | 0.196 | |
| Abnormal Glucose | | | | | | | | | | |
| Estrogen-progestin | 0.92 (0.36-2.31) | 0.854 | 0.75 (0.27-2.06) | 0.578 | 0.71 (0.25-2.03) | 0.519 | 0.76 (0.28-2.08) | 0.591 | 0.71 (0.25-2.02) | 0.519 |
| EAA | 1.01 (0.98-1.04) | 0.639 | 1.01 (0.98-1.03) | 0.655 | 1.00 (0.98-1.02) | 0.863 | 1.05 (1.01-1.10) | 0.028 | 1.06 (1.01-1.10) | 0.012 |
| Estrogen-progestin× EAA | 1.12 (0.89-1.42) | 0.335 | 1.08 (0.81-1.43) | 0.618 | 1.14 (1.00-1.31) | 0.055 | 1.08 (0.92-1.27) | 0.319 | 1.08 (0.93-1.26) | 0.320 |
| PHA P value | 0.456 | | 0.500 | | 0.216 | | 0.425 | | 0.401 | |

All models were stratified by age and adjusted for race, education level, PIR, BMI, smoking status, alcohol consumption, hypertension, hyperlipidemia, cardiovascular disease, chronic kidney disease, and cancer. The three-way interaction terms (Estrogen-progestin combined therapy × EAA × glycemic status) represent how the relationship between Estrogen-progestin combined therapy and mortality risk is modified jointly by EAA and glycemic status. EAA, epigenetic age acceleration; HR, hazard ratio; CI, confidence interval; PHA, proportional hazards assumption; PIR, poverty-to-income ratio; BMI, body mass index.

**Table S18.** Sensitivity analysis: three-way interaction of HRT, EAA and glycemic status, along with glycemic-stratified two-way interaction analysis on all-cause mortality risk among participants aged 65 years or older.

| Variable | HorvathAA | | HannumAA | | PhenoAA | | GrimAA | | Grim2AA | |
| --- | --- | --- | --- | --- | --- | --- | --- | --- | --- | --- |
| Three-way interaction/HR (95% CI), P value | | | | | | | | | | |
| HRT × EAA ×  Abnormal Glucose | 1.05 (0.88-1.25) | 0.601 | 1.03 (0.84-1.27) | 0.768 | 1.00 (0.88-1.15) | 0.959 | 1.09 (0.90-1.31) | 0.370 | 1.08 (0.91-1.27) | 0.399 |
| PHA P value | 0.927 | | 0.859 | | 0.907 | | 0.914 | | 0.934 | |
| Two-way interaction stratified by glycemic status/ HR (95% CI), P value | | | | | | | | | | |
| Normal Glucose | | | | | | | | | | |
| HRT | 1.29 (0.61-2.69) | 0.506 | 1.13 (0.54-2.39) | 0.741 | 1.18 (0.57-2.41) | 0.659 | 1.13 (0.55-2.34) | 0.736 | 1.05 (0.51-2.17) | 0.897 |
| EAA | 1.06 (0.99-1.12) | 0.077 | 1.06 (1.00-1.11) | 0.048 | 1.07 (1.02-1.12) | 0.008 | 1.22 (1.12-1.33) | <0.001 | 1.21 (1.11-1.32) | <0.001 |
| HRT × EAA | 1.01 (0.88-1.15) | 0.935 | 0.96 (0.83-1.11) | 0.587 | 0.97 (0.85-1.10) | 0.609 | 0.87 (0.74-1.02) | 0.081 | 0.88 (0.76-1.01) | 0.077 |
| PHA P value | 0.259 | | 0.320 | | 0.215 | | 0.427 | | 0.462 | |
| Abnormal Glucose | | | | | | | | | | |
| HRT | 0.49 (0.27-0.87) | 0.016 | 0.49 (0.27-0.89) | 0.018 | 0.51 (0.29-0.91) | 0.023 | 0.53 (0.29-0.96) | 0.037 | 0.55 (0.30-1.00) | 0.048 |
| EAA | 1.00 (0.96-1.03) | 0.899 | 1.00 (0.97-1.04) | 0.859 | 1.01 (0.98-1.03) | 0.551 | 1.05 (0.99-1.11) | 0.127 | 1.06 (1.00-1.11) | 0.045 |
| HRT × EAA | 1.10 (0.96-1.26) | 0.158 | 1.04 (0.87-1.25) | 0.643 | 1.01 (0.93-1.10) | 0.761 | 0.99 (0.87-1.13) | 0.896 | 0.99 (0.88-1.11) | 0.818 |
| PHA P value | 0.750 | | 0.830 | | 0.975 | | 0.976 | | 0.977 | |

All models were stratified by age and adjusted for race, education level, PIR, BMI, smoking status, alcohol consumption, hypertension, hyperlipidemia, cardiovascular disease, chronic kidney disease, and cancer. The three-way interaction terms (HRT × EAA × glycemic status) represent how the relationship between HRT and mortality risk is modified jointly by EAA and glycemic status. HRT, hormone replacement therapy; EAA, epigenetic age acceleration; HR, hazard ratio; CI, confidence interval; PHA, proportional hazards assumption; PIR, poverty-to-income ratio; BMI, body mass index.

**Table S19.** Sensitivity analysis: three-way interaction of HRT, EAA and glycemic status, along with glycemic-stratified two-way interaction analysis on all-cause mortality risk among participants aged younger than 65 years.

| Variable | HorvathAA | | HannumAA | | PhenoAA | | GrimAA | | Grim2AA | |
| --- | --- | --- | --- | --- | --- | --- | --- | --- | --- | --- |
| Three-way interaction/HR (95% CI), P value | | | | | | | | | | |
| HRT × EAA ×  Abnormal Glucose | 1.21 (0.97-1.50) | 0.088 | 1.08 (0.89-1.32) | 0.423 | 1.21 (1.04-1.40) | 0.014 | 1.11 (0.92-1.34) | 0.264 | 1.09 (0.92-1.30) | 0.319 |
| PHA P value | 0.161 | | 0.073 | | 0.043 | | 0.096 | | 0.098 | |
| Two-way interaction stratified by glycemic status/ HR (95% CI), P value | | | | | | | | | | |
| Normal Glucose | | | | | | | | | | |
| HRT | 0.49 (0.22-1.13) | 0.094 | 0.41 (0.17-0.97) | 0.042 | 0.49 (0.20-1.18) | 0.111 | 0.44 (0.18-1.05) | 0.064 | 0.43 (0.18-1.03) | 0.059 |
| EAA | 1.09 (1.00-1.19) | 0.057 | 1.09 (1.00-1.18) | 0.042 | 1.14 (1.05-1.24) | 0.003 | 1.06 (0.95-1.17) | 0.305 | 1.07 (0.98-1.17) | 0.143 |
| HRT × EAA | 0.87 (0.73-1.04) | 0.121 | 0.95 (0.80-1.12) | 0.539 | 0.91 (0.79-1.04) | 0.169 | 1.00 (0.86-1.16) | 0.975 | 1.00 (0.87-1.15) | 0.996 |
| PHA P value | 0.045 | | 0.074 | | 0.052 | | 0.077 | | 0.070 | |
| Abnormal Glucose | | | | | | | | | | |
| HRT | 0.75 (0.35-1.62) | 0.460 | 0.76 (0.35-1.65) | 0.494 | 0.72 (0.32-1.63) | 0.436 | 0.75 (0.33-1.68) | 0.482 | 0.74 (0.33-1.68) | 0.475 |
| EAA | 1.03 (0.97-1.09) | 0.287 | 1.02 (0.97-1.08) | 0.349 | 0.99 (0.95-1.03) | 0.751 | 1.06 (0.97-1.15) | 0.191 | 1.05 (0.97-1.13) | 0.220 |
| HRT × EAA | 1.12 (0.96-1.30) | 0.162 | 1.06 (0.93-1.22) | 0.366 | 1.12 (1.01-1.23) | 0.024 | 1.06 (0.93-1.21) | 0.390 | 1.05 (0.93-1.19) | 0.405 |
| PHA P value | 0.875 | | 0.872 | | 0.451 | | 0.757 | | 0.699 | |

All models were stratified by age and adjusted for race, education level, PIR, BMI, smoking status, alcohol consumption, hypertension, hyperlipidemia, cardiovascular disease, chronic kidney disease, and cancer. The three-way interaction terms (HRT × EAA × glycemic status) represent how the relationship between HRT and mortality risk is modified jointly by EAA and glycemic status. HRT, hormone replacement therapy; EAA, epigenetic age acceleration; HR, hazard ratio; CI, confidence interval; PHA, proportional hazards assumption; PIR, poverty-to-income ratio; BMI, body mass index.

**Table S20.** Three-way interaction of HRT, EAA and glycemic status, along with glycemic-stratified two-way interaction analysis on cardiovascular mortality risk.

| Variable | HorvathAA | | HannumAA | | PhenoAA | | GrimAA | | Grim2AA | |
| --- | --- | --- | --- | --- | --- | --- | --- | --- | --- | --- |
| Three-way interaction/HR (95% CI), P value | | | | | | | | | | |
| HRT × EAA ×  Abnormal Glucose | 1.07 (0.82-1.39) | 0.624 | 1.02 (0.80-1.31) | 0.850 | 1.07 (0.89-1.28) | 0.480 | 1.15 (0.87-1.51) | 0.336 | 1.11 (0.86-1.42) | 0.437 |
| PHA P value | 0.586 | | 0.402 | | 0.607 | | 0.404 | | 0.362 | |
| Two-way interaction stratified by glycemic status/ HR (95% CI), P value | | | | | | | | | | |
| Normal Glucose | | | | | | | | | | |
| HRT | 0.55 (0.20-1.50) | 0.243 | 0.53 (0.20-1.45) | 0.217 | 0.51 (0.18-1.42) | 0.198 | 0.55 (0.19-1.54) | 0.254 | 0.51 (0.18-1.46) | 0.210 |
| EAA | 1.02 (0.93-1.11) | 0.728 | 1.01 (0.93-1.09) | 0.881 | 1.02 (0.96-1.10) | 0.492 | 1.11 (0.99-1.24) | 0.084 | 1.07 (0.96-1.19) | 0.212 |
| HRT × EAA | 1.02 (0.83-1.26) | 0.827 | 1.01 (0.84-1.21) | 0.932 | 0.95 (0.82-1.10) | 0.501 | 0.90 (0.73-1.10) | 0.295 | 0.91 (0.75-1.11) | 0.346 |
| PHA P value | 0.146 | | 0.098 | | 0.114 | | 0.187 | | 0.181 | |
| Abnormal Glucose | | | | | | | | | | |
| HRT | 0.45 (0.19-1.06) | 0.069 | 0.42 (0.18-1.01) | 0.052 | 0.41 (0.17-1.01) | 0.052 | 0.42 (0.16-1.06) | 0.067 | 0.43 (0.17-1.07) | 0.070 |
| EAA | 1.00 (0.95-1.05) | 0.907 | 1.00 (0.95-1.05) | 0.948 | 0.98 (0.94-1.02) | 0.353 | 1.03 (0.95-1.12) | 0.530 | 1.02 (0.95-1.10) | 0.578 |
| HRT × EAA | 1.14 (0.94-1.38) | 0.198 | 1.02 (0.83-1.25) | 0.851 | 1.01 (0.89-1.15) | 0.864 | 1.03 (0.84-1.25) | 0.808 | 1.00 (0.84-1.20) | 0.966 |
| PHA P value | 0.472 | | 0.305 | | 0.484 | | 0.200 | | 0.214 | |

All models were stratified by age and adjusted for race, education level, PIR, BMI, smoking status, alcohol consumption, hypertension, hyperlipidemia, cardiovascular disease, chronic kidney disease, and cancer. The three-way interaction terms (HRT × EAA × glycemic status) represent how the relationship between HRT and mortality risk is modified jointly by EAA and glycemic status. HRT, hormone replacement therapy; EAA, epigenetic age acceleration; HR, hazard ratio; CI, confidence interval; PHA, proportional hazards assumption; PIR, poverty-to-income ratio; BMI, body mass index.

**Table S21.** Sensitivity analysis: three-way interaction of HRT, EAA and glycemic status, along with glycemic-stratified two-way interaction analysis on cardiovascular mortality risk excluding participants with less than 2 years of follow-up.

| Variable | HorvathAA | | HannumAA | | PhenoAA | | GrimAA | | Grim2AA | |
| --- | --- | --- | --- | --- | --- | --- | --- | --- | --- | --- |
| Three-way interaction/HR (95% CI), P value | | | | | | | | | | |
| HRT × EAA ×  Abnormal Glucose | 1.06 (0.81-1.38) | 0.688 | 1.02 (0.79-1.31) | 0.904 | 1.06 (0.88-1.28) | 0.526 | 1.12 (0.85-1.49) | 0.416 | 1.08 (0.84-1.40) | 0.531 |
| PHA P value | 0.449 | | 0.243 | | 0.452 | | 0.285 | | 0.259 | |
| Two-way interaction stratified by glycemic status/ HR (95% CI), P value | | | | | | | | | | |
| Normal Glucose | | | | | | | | | | |
| HRT | 0.61 (0.22-1.68) | 0.339 | 0.61 (0.22-1.67) | 0.334 | 0.56 (0.20-1.60) | 0.281 | 0.61 (0.21-1.73) | 0.348 | 0.57 (0.19-1.65) | 0.299 |
| EAA | 1.00 (0.91-1.10) | 0.927 | 1.00 (0.92-1.08) | 0.907 | 1.02 (0.95-1.09) | 0.596 | 1.11 (0.98-1.26) | 0.086 | 1.08 (0.96-1.21) | 0.206 |
| HRT × EAA | 1.03 (0.83-1.29) | 0.764 | 1.04 (0.85-1.26) | 0.707 | 0.96 (0.82-1.12) | 0.593 | 0.91 (0.74-1.12) | 0.371 | 0.92 (0.76-1.12) | 0.424 |
| PHA P value | 0.147 | | 0.115 | | 0.096 | | 0.167 | | 0.161 | |
| Abnormal Glucose | | | | | | | | | | |
| HRT | 0.45 (0.19-1.07) | 0.070 | 0.42 (0.17-1.01) | 0.052 | 0.41 (0.17-1.01) | 0.053 | 0.42 (0.16-1.06) | 0.067 | 0.43 (0.17-1.08) | 0.073 |
| EAA | 1.00 (0.95-1.05) | 0.961 | 1.00 (0.95-1.05) | 0.975 | 0.98 (0.94-1.02) | 0.324 | 1.02 (0.94-1.12) | 0.576 | 1.02 (0.95-1.10) | 0.590 |
| HRT × EAA | 1.14 (0.94-1.39) | 0.187 | 1.02 (0.83-1.25) | 0.850 | 1.02 (0.89-1.16) | 0.820 | 1.03 (0.84-1.26) | 0.812 | 1.00 (0.84-1.20) | 0.999 |
| PHA P value | 0.128 | | 0.064 | | 0.148 | | 0.030 | | 0.037 | |

All models were stratified by age and adjusted for race, education level, PIR, BMI, smoking status, alcohol consumption, hypertension, hyperlipidemia, cardiovascular disease, chronic kidney disease, and cancer. The three-way interaction terms (HRT × EAA × glycemic status) represent how the relationship between HRT and mortality risk is modified jointly by EAA and glycemic status. HRT, hormone replacement therapy; EAA, epigenetic age acceleration; HR, hazard ratio; CI, confidence interval; PHA, proportional hazards assumption; PIR, poverty-to-income ratio; BMI, body mass index.

**Table S22.** Sensitivity analysis: three-way interaction of estrogen-only therapy, EAA and glycemic status, along with glycemic-stratified two-way interaction analysis on cardiovascular mortality risk.

| Variable | HorvathAA | | HannumAA | | PhenoAA | | GrimAA | | Grim2AA | |
| --- | --- | --- | --- | --- | --- | --- | --- | --- | --- | --- |
| Three-way interaction/HR (95% CI), P value | | | | | | | | | | |
| Estrogen-only × EAA × Abnormal Glucose | 1.28 (0.91-1.82) | 0.158 | 1.08 (0.82-1.41) | 0.604 | 1.10 (0.89-1.37) | 0.361 | 1.09 (0.79-1.51) | 0.603 | 1.05 (0.79-1.40) | 0.742 |
| PHA P value | 0.685 | | 0.444 | | 0.717 | | 0.461 | | 0.434 | |
| Two-way interaction stratified by glycemic status/ HR (95% CI), P value | | | | | | | | | | |
| Normal Glucose | | | | | | | | | | |
| Estrogen-only | 0.39 (0.11-1.40) | 0.148 | 0.42 (0.12-1.43) | 0.165 | 0.42 (0.12-1.44) | 0.168 | 0.51 (0.16-1.59) | 0.244 | 0.48 (0.15-1.52) | 0.214 |
| EAA | 1.01 (0.92-1.11) | 0.828 | 1.00 (0.93-1.09) | 0.949 | 1.02 (0.95-1.09) | 0.544 | 1.10 (0.98-1.23) | 0.110 | 1.06 (0.95-1.18) | 0.285 |
| Estrogen-only × EAA | 0.86 (0.63-1.18) | 0.364 | 0.93 (0.74-1.16) | 0.512 | 0.93 (0.77-1.11) | 0.416 | 0.95 (0.77-1.18) | 0.660 | 0.96 (0.78-1.18) | 0.699 |
| PHA P value | 0.179 | | 0.091 | | 0.154 | | 0.139 | | 0.137 | |
| Abnormal Glucose | | | | | | | | | | |
| Estrogen-only | 0.34 (0.12-0.95) | 0.040 | 0.34 (0.13-0.95) | 0.039 | 0.35 (0.12-0.96) | 0.041 | 0.33 (0.11-1.01) | 0.053 | 0.35 (0.12-1.02) | 0.055 |
| EAA | 1.00 (0.95-1.05) | 0.967 | 1.00 (0.95-1.05) | 0.999 | 0.98 (0.94-1.02) | 0.360 | 1.03 (0.95-1.12) | 0.485 | 1.02 (0.95-1.10) | 0.571 |
| Estrogen-only × EAA | 1.21 (0.98-1.50) | 0.080 | 0.98 (0.81-1.19) | 0.856 | 1.02 (0.88-1.18) | 0.839 | 1.05 (0.82-1.35) | 0.703 | 1.00 (0.81-1.24) | 0.966 |
| PHA P value | 0.316 | | 0.166 | | 0.303 | | 0.121 | | 0.132 | |

All models were stratified by age and adjusted for race, education level, PIR, BMI, smoking status, alcohol consumption, hypertension, hyperlipidemia, cardiovascular disease, chronic kidney disease, and cancer. The three-way interaction terms (Estrogen-only therapy × EAA × glycemic status) represent how the relationship between estrogen-only therapy and mortality risk is modified jointly by EAA and glycemic status. EAA, epigenetic age acceleration; HR, hazard ratio; CI, confidence interval; PHA, proportional hazards assumption; PIR, poverty-to-income ratio; BMI, body mass index.

**Table S23.** Sensitivity analysis: three-way interaction of estrogen-progestin combined therapy, EAA and glycemic status, along with glycemic-stratified two-way interaction analysis on cardiovascular mortality risk.

| Variable | HorvathAA | | HannumAA | | PhenoAA | | GrimAA | | Grim2AA | |
| --- | --- | --- | --- | --- | --- | --- | --- | --- | --- | --- |
| Three-way interaction/HR (95% CI), P value | | | | | | | | | | |
| Estrogen-progestin× EAA × Abnormal Glucose | 0.73 (0.39-1.39) | 0.342 | 0.91 (0.51-1.63) | 0.748 | 0.94 (0.63-1.41) | 0.776 | 1.30 (0.81-2.07) | 0.279 | 1.24 (0.80-1.93) | 0.341 |
| PHA P value | 0.788 | | 0.580 | | 0.779 | | 0.611 | | 0.546 | |
| Two-way interaction stratified by glycemic status/ HR (95% CI), P value | | | | | | | | | | |
| Normal Glucose | | | | | | | | | | |
| Estrogen-progestin | 0.56 (0.11-2.87) | 0.487 | 0.49 (0.09-2.75) | 0.419 | 0.56 (0.11-2.88) | 0.487 | 0.31 (0.03-2.99) | 0.314 | 0.31 (0.03-2.92) | 0.303 |
| EAA | 1.01 (0.92-1.11) | 0.785 | 1.00 (0.92-1.08) | 0.957 | 1.01 (0.94-1.09) | 0.762 | 1.10 (0.97-1.24) | 0.144 | 1.06 (0.95-1.18) | 0.329 |
| Estrogen-progestin× EAA | 1.26 (0.91-1.76) | 0.167 | 1.11 (0.78-1.56) | 0.566 | 1.01 (0.79-1.29) | 0.940 | 0.71 (0.43-1.16) | 0.168 | 0.78 (0.51-1.18) | 0.239 |
| PHA P value | 0.385 | | 0.209 | | 0.323 | | 0.365 | | 0.343 | |
| Abnormal Glucose | | | | | | | | | | |
| Estrogen-progestin | 0.71 (0.07-7.08) | 0.774 | 0.92 (0.17-5.02) | 0.923 | 1.01 (0.20-5.05) | 0.995 | 1.05 (0.23-4.85) | 0.946 | 1.04 (0.22-4.99) | 0.964 |
| EAA | 1.00 (0.96-1.05) | 0.900 | 1.00 (0.96-1.05) | 0.969 | 0.98 (0.95-1.02) | 0.387 | 1.03 (0.94-1.12) | 0.558 | 1.02 (0.94-1.10) | 0.610 |
| Estrogen-progestin× EAA | 0.86 (0.49-1.52) | 0.606 | 1.11 (0.70-1.75) | 0.650 | 0.99 (0.73-1.33) | 0.941 | 0.99 (0.74-1.33) | 0.956 | 1.00 (0.75-1.34) | 0.995 |
| PHA P value | 0.704 | | 0.546 | | 0.740 | | 0.344 | | 0.324 | |

All models were stratified by age and adjusted for race, education level, PIR, BMI, smoking status, alcohol consumption, hypertension, hyperlipidemia, cardiovascular disease, chronic kidney disease, and cancer. The three-way interaction terms (Estrogen-progestin combined therapy × EAA × glycemic status) represent how the relationship between estrogen-progestin combined therapy and mortality risk is modified jointly by EAA and glycemic status. EAA, epigenetic age acceleration; HR, hazard ratio; CI, confidence interval; PHA, proportional hazards assumption; PIR, poverty-to-income ratio; BMI, body mass index.

**Table S24.** Sensitivity analysis: three-way interaction of HRT, EAA and glycemic status, along with glycemic-stratified two-way interaction analysis on cardiovascular mortality risk among participants aged 65 years or older.

| Variable | HorvathAA | | HannumAA | | PhenoAA | | GrimAA | | Grim2AA | |
| --- | --- | --- | --- | --- | --- | --- | --- | --- | --- | --- |
| Three-way interaction/HR (95% CI), P value | | | | | | | | | | |
| HRT × EAA ×  Abnormal Glucose | 1.19 (0.83-1.70) | 0.332 | 1.10 (0.69-1.74) | 0.690 | 1.00 (0.74-1.36) | 0.9775 | 1.22 (0.77-1.92) | 0.401 | 1.17 (0.78-1.77) | 0.442 |
| PHA P value | 0.701 | | 0.727 | | 0.641 | | 0.308 | | 0.354 | |
| Two-way interaction stratified by glycemic status/ HR (95% CI), P value | | | | | | | | | | |
| Normal Glucose | | | | | | | | | | |
| HRT | 0.62 (0.13-2.92) | 0.546 | 0.79 (0.17-3.78) | 0.771 | 0.65 (0.13-3.16) | 0.589 | 0.55 (0.11-2.74) | 0.465 | 0.51 (0.09-2.77) | 0.434 |
| EAA | 1.00 (0.89-1.12) | 0.940 | 0.98 (0.88-1.10) | 0.763 | 1.01 (0.92-1.10) | 0.879 | 1.16 (0.98-1.39) | 0.089 | 1.11 (0.93-1.31) | 0.247 |
| HRT × EAA | 1.01 (0.72-1.42) | 0.955 | 1.12 (0.81-1.56) | 0.486 | 1.02 (0.78-1.34) | 0.898 | 0.90 (0.63-1.27) | 0.536 | 0.91 (0.65-1.26) | 0.557 |
| PHA P value | 0.248 | | 0.169 | | 0.192 | | 0.146 | | 0.147 | |
| Abnormal Glucose | | | | | | | | | | |
| HRT | 0.33 (0.11-1.05) | 0.060 | 0.29 (0.09-0.98) | 0.046 | 0.29 (0.08-1.03) | 0.056 | 0.26 (0.07-0.93) | 0.038 | 0.28 (0.08-0.94) | 0.039 |
| EAA | 0.98 (0.93-1.04) | 0.501 | 0.98 (0.92-1.04) | 0.425 | 0.98 (0.94-1.03) | 0.484 | 1.01 (0.90-1.14) | 0.843 | 1.03 (0.93-1.14) | 0.584 |
| HRT × EAA | 1.31 (1.02-1.67) | 0.034 | 1.20 (0.76-1.89) | 0.427 | 1.04 (0.83-1.31) | 0.714 | 1.11 (0.75-1.64) | 0.597 | 1.02 (0.75-1.39) | 0.888 |
| PHA P value | 0.497 | | 0.411 | | 0.378 | | 0.097 | | 0.112 | |

All models were stratified by age and adjusted for race, education level, PIR, BMI, smoking status, alcohol consumption, hypertension, hyperlipidemia, cardiovascular disease, chronic kidney disease, and cancer. The three-way interaction terms (HRT × EAA × glycemic status) represent how the relationship between HRT and mortality risk is modified jointly by EAA and glycemic status. HRT, hormone replacement therapy; EAA, epigenetic age acceleration; HR, hazard ratio; CI, confidence interval; PHA, proportional hazards assumption; PIR, poverty-to-income ratio; BMI, body mass index.

**Table S25.** Sensitivity analysis: three-way interaction of HRT, EAA and glycemic status, along with glycemic-stratified two-way interaction analysis on cardiovascular mortality risk among participants aged younger than 65 years.

| Variable | HorvathAA | | HannumAA | | PhenoAA | | GrimAA | | Grim2AA | |
| --- | --- | --- | --- | --- | --- | --- | --- | --- | --- | --- |
| Three-way interaction/HR (95% CI), P value | | | | | | | | | | |
| HRT × EAA ×  Abnormal Glucose | 0.94 (0.63-1.42) | 0.783 | 0.92 (0.60-1.40) | 0.685 | 1.10 (0.81-1.49) | 0.539 | 1.15 (0.79-1.68) | 0.456 | 1.10 (0.78-1.56) | 0.591 |
| PHA P value | 0.772 | | 0.663 | | 0.804 | | 0.847 | | 0.798 | |
| Two-way interaction stratified by glycemic status/ HR (95% CI), P value | | | | | | | | | | |
| Normal Glucose | | | | | | | | | | |
| HRT | 0.20 (0.02-1.96) | 0.167 | 0.15 (0.01-1.72) | 0.127 | 0.18 (0.02-1.87) | 0.152 | 0.19 (0.02-2.10) | 0.176 | 0.18 (0.05-0.71) | 0.014 |
| EAA | 1.07 (0.89-1.28) | 0.483 | 1.13 (0.93-1.38) | 0.206 | 1.11 (0.95-1.28) | 0.190 | 1.18 (0.97-1.45) | 0.101 | 1.09 (0.99-1.20) | 0.066 |
| HRT × EAA | 1.12 (0.76-1.66) | 0.571 | 1.01 (0.71-1.42) | 0.974 | 1.01 (0.76-1.34) | 0.938 | 1.00 (0.69-1.44) | 0.988 | 1.05 (0.85-1.31) | 0.627 |
| PHA P value | NA | | 0.129 | | 0.182 | | 0.045 | | 0.062 | |
| Abnormal Glucose | | | | | | | | | | |
| HRT | 1.58 (0.33-7.49) | 0.566 | 1.70 (0.32-8.93) | 0.530 | 1.44 (0.30-6.81) | 0.645 | 1.62 (0.31-8.41) | 0.565 | 1.54 (0.31-7.72) | 0.598 |
| EAA | 1.14 (1.00-1.29) | 0.043 | 1.17 (1.04-1.31) | 0.009 | 1.03 (0.94-1.13) | 0.489 | 1.13 (0.97-1.32) | 0.104 | 1.07 (0.94-1.23) | 0.293 |
| HRT × EAA | 0.90 (0.65-1.25) | 0.525 | 0.84 (0.62-1.14) | 0.262 | 0.95 (0.76-1.20) | 0.687 | 0.97 (0.74-1.27) | 0.835 | 0.98 (0.77-1.25) | 0.883 |
| PHA P value | 0.498 | | 0.369 | | 0.652 | | 0.625 | | 0.666 | |

All models were stratified by age and adjusted for race, education level, PIR, BMI, smoking status, alcohol consumption, hypertension, hyperlipidemia, cardiovascular disease, chronic kidney disease, and cancer. The three-way interaction terms (HRT × EAA × glycemic status) represent how the relationship between HRT and mortality risk is modified jointly by EAA and glycemic status. HRT, hormone replacement therapy; EAA, epigenetic age acceleration; HR, hazard ratio; CI, confidence interval; PHA, proportional hazards assumption; PIR, poverty-to-income ratio; BMI, body mass index.

**Table S26.** Comparison of baseline characteristics between included and excluded postmenopausal women.

| **Variable** | **Included group (n=673)** | **Excluded group (n=843)** | **P value** |
| --- | --- | --- | --- |
| Age, median (Q1–Q3) |  |  |  |
| Chronological Age | 63.00 (57.00, 69.00) | 62.00 (56.00, 68.00) | 0.015 |
| Race |  |  | <0.001 |
| Non-Hispanic White | 242 (36.0%) | 493 (58.5%) |  |
| Non-Hispanic Black | 161 (23.9%) | 128 (15.2%) |  |
| Mexican American | 195 (29.0%) | 169 (20.0%) |  |
| Hispanic and Other | 75 (11.1%) | 53 (6.3%) |  |
| Education |  |  | <0.001 |
| Less than High School | 306 (45.5%) | 299 (35.5%) |  |
| High School | 152 (22.6%) | 202 (24.0%) |  |
| More than High School | 215 (31.9%) | 342 (40.6%) |  |
| BMI (kg/m^2^) |  |  | 0.049 |
| <25 | 179 (26.6%) | 237 (28.1%) |  |
| 25~30 | 198 (29.4%) | 286 (33.9%) |  |
| >30 | 296 (44.0%) | 320 (38.0%) |  |
| PIR |  |  | 0.026 |
| <1.3 | 214 (31.8%) | 215 (25.5%) |  |
| 1.3~3.5 | 249 (37.0%) | 343 (40.7%) |  |
| >3.5 | 210 (31.2%) | 285 (33.8%) |  |
| Glycemic Status |  |  | <0.001 |
| Abnormal | 369 (54.8%) | 379 (45.0%) |  |
| Normal | 304 (45.2%) | 464 (55.0%) |  |
| Hypertension |  |  | 0.025 |
| Yes | 518 (77.0%) | 605 (71.8%) |  |
| No | 155 (23.0%) | 238 (28.2%) |  |
| Hyperlipidemia |  |  | 0.030 |
| Yes | 591 (87.8%) | 706 (83.7%) |  |
| No | 82 (12.2%) | 137 (16.3%) |  |
| Cardiovascular Diseases |  |  | 0.019 |
| Yes | 97 (14.4%) | 87 (10.3%) |  |
| No | 576 (85.6%) | 756 (89.7%) |  |
| Chronic Kidney Disease |  |  | 0.022 |
| Yes | 156 (23.2%) | 154 (18.3%) |  |
| No | 517 (76.8%) | 689 (81.7%) |  |
| Cancer |  |  | 0.325 |
| Yes | 84 (12.5%) | 121 (14.4%) |  |
| No | 589 (87.5%) | 722 (85.6%) |  |
| Smoking |  |  | 0.022 |
| Never | 384 (57.1%) | 493 (58.5%) |  |
| Before | 193 (28.7%) | 197 (23.4%) |  |
| Now | 96 (14.3%) | 153 (18.1%) |  |
| Drinking |  |  | 0.005 |
| Yes | 302 (44.9%) | 440 (52.2%) |  |
| No | 371 (55.1%) | 403 (47.8%) |  |

BMI, body mass index; PIR, poverty-to-income ratio.

**Figure S1.** The leave-one-out plot of Mendelian randomization analysis results.

A-D: the exposure is ESR1; the outcome is GrimAge acceleration (A), HannumAge acceleration (B), HorvathAge acceleration (C), PhenoAge acceleration (D). E-H: the exposure is ESR2; the outcome is GrimAge acceleration (E), HannumAge acceleration (F), HorvathAge acceleration (G), PhenoAge acceleration (H). ESR1, estrogen receptor alpha; ESR2, estrogen receptor beta.

**Figure S2.** The forest plot of Mendelian randomization analysis results.

A-D: the exposure is ESR1; the outcome is GrimAge acceleration (A), HannumAge acceleration (B), HorvathAge acceleration (C), PhenoAge acceleration (D). E-H: the exposure is ESR2; the outcome is GrimAge acceleration (E), HannumAge acceleration (F), HorvathAge acceleration (G), PhenoAge acceleration (H). ESR1, estrogen receptor alpha; ESR2, estrogen receptor beta.

**Figure S3.** The funnel plot of Mendelian randomization analysis results.

A-D: the exposure is ESR1; the outcome is GrimAge acceleration (A), HannumAge acceleration (B), HorvathAge acceleration (C), PhenoAge acceleration (D). E-H: the exposure is ESR2; the outcome is GrimAge acceleration (E), HannumAge acceleration (F), HorvathAge acceleration (G), PhenoAge acceleration (H). ESR1, estrogen receptor alpha; ESR2, estrogen receptor beta.

**Figure S4.** The scatter plot of Mendelian randomization analysis results.

A-D: the exposure is ESR1; the outcome is GrimAge acceleration (A), HannumAge acceleration (B), HorvathAge acceleration (C), PhenoAge acceleration (D). E-H: the exposure is ESR2; the outcome is GrimAge acceleration (E), HannumAge acceleration (F), HorvathAge acceleration (G), PhenoAge acceleration (H). ESR1, estrogen receptor alpha; ESR2, estrogen receptor beta.
